# Mechanisms and Therapeutic Advances of Natural Products Regulating Non-Coding RNAs in Cancer

**DOI:** 10.3390/ph19101560

**Published:** 2026-10-01

**Authors:** Qianhui You, Runfang Pan, Haidong Guo, Baonian Liu

**Affiliations:** Department of Anatomy, School of Integrative Medicine, Shanghai University of Traditional Chinese Medicine, Shanghai 201203, China; nk31ultraver@outlook.com (Q.Y.); many_many_circles@163.com (R.P.)

**Keywords:** non-coding RNAs, natural products, cancer, therapeutic target, targeted therapy

## Abstract

Non-coding RNAs (ncRNAs), including microRNAs, long non-coding RNAs, circular RNAs, tRNA-derived fragments, PIWI-interacting RNAs, and small nucleolar RNAs, have been identified as crucial regulators of gene expression and key drivers of cancer progression. Natural products, owing to their structural diversity and multi-targeting capacity, represent a promising reservoir for anticancer drug discovery through the modulation of these ncRNA networks. This review provides a comprehensive overview of the regulatory roles of natural products on ncRNAs in cancer. Compared with previous reviews that focus on individual ncRNA classes or single natural product categories, this review provides an integrated framework linking ncRNA biology, natural product pharmacology, and therapeutic delivery in cancer. First, the biogenesis, classification, and molecular functions of major ncRNA classes are systematically described, and the dysregulation of each ncRNA class across various cancer types and their contributions to tumor pathogenesis are then discussed. Subsequently, the modulation of these ncRNA networks by representative natural products, including polyphenols, flavonoids, terpenoids, and alkaloids, to influence multiple hallmarks of tumor progression, encompassing cell proliferation, programmed cell death, metastatic dissemination, tumor microenvironment remodeling, and therapeutic resistance, is reviewed. The potential of ncRNAs as diagnostic, prognostic, and pharmacodynamic biomarkers in the context of natural product-based therapy is discussed, together with emerging nanoparticle-based delivery strategies for co-delivering natural products and ncRNA therapeutics; however, most of the evidence summarized here remains preclinical, and clinical translation will require rigorous pharmacological and clinical validation. Finally, we address current challenges and future perspectives for the clinical translation of natural product–ncRNA-based anticancer strategies.

## 1. Introduction

Cancer remains one of the leading causes of mortality worldwide and poses a major burden on global health. Globally, GLOBOCAN 2024 estimated approximately 20.6 million new cancer cases and 9.8 million cancer deaths worldwide in 2024, corresponding to about one in five people developing cancer during their lifetime and one in nine men and one in thirteen women dying from the disease [1]. Lung cancer remained the most commonly diagnosed cancer, with nearly 2.6 million new cases (12.8% of all cases), followed by female breast (11.8%), colorectal (9.9%), prostate (7.5%), and stomach (4.7%) cancers, and it was also the leading cause of cancer death (approximately 1.9 million deaths, 19.1%), followed by colorectal (9.4%), liver (7.5%), female breast (7.1%), and stomach (6.6%) cancers [2]. Despite great advances in surgery, chemotherapy, targeted therapies, immunotherapies, and radiotherapy in the last decades, the clinical management of cancer still faces challenges, including acquired drug resistance, significant intra-tumor heterogeneity, and metastatic dissemination [3]. These obstacles highlight the need for advanced therapeutic approaches that are more effective and less toxic. In this context, natural products have garnered increasing attention as a promising option for anticancer drug discovery, due to their structural diversity, multi-targeting capacity, safety profiles, and ability to modulate a broad range of cellular signaling [4]. Representative polyphenols, including curcumin, resveratrol, and epigallocatechin-3-gallate, flavonoids such as baicalein and quercetin, and terpenoids, including ginsenosides and artemisinin derivatives, have shown potent antitumor activities against a wide spectrum of malignancies through diverse molecular mechanisms [5]. Furthermore, integrating natural products with advanced drug delivery platforms, especially biomimetic nanoparticle- and exosome-based systems, has emerged as a promising strategy to enhance their efficacy and bioavailability [6,7]. However, despite these advances, the precise molecular mechanisms through which natural products exert their anticancer effects remain incompletely understood.

Non-coding RNAs (ncRNAs) are a heterogeneous group of RNA transcripts that function as critical modulators of gene expression at the transcriptional, post-transcriptional, and epigenetic levels [8]. According to the size of the transcript, structural features, and biogenesis pathways, ncRNAs are generally divided into several major classes. MicroRNAs (miRNAs) are small ncRNAs of about 19 to 25 nucleotides in length that direct the RNA-induced silencing complex (RISC) to target mRNAs through complementary base pairing, mainly resulting in translational repression or mRNA destabilisation [9]. Long non-coding RNAs (lncRNAs) are transcripts longer than 200 nucleotides that modulate chromatin structure, transcriptional processes, and post-transcriptional activities via diverse mechanisms, including molecular scaffolding, transcriptional decoy activity, and competing endogenous RNA (ceRNA) networks [10,11]. Circular RNAs (circRNAs) represent a novel class of RNAs with a covalently closed loop structure generated by back-splicing of pre-mRNA transcripts. circRNAs are highly stable, function as miRNA sponges, protein scaffolds, transcriptional regulators, and even templates for cap-independent translation mediated by N6-methyladenosine modifications or internal ribosome entry sites [12,13,14]. In addition to these well-studied classes, other regulatory ncRNAs such as tRNA-derived fragments (tsRNAs), PIWI-interacting RNAs (piRNAs), and small nucleolar RNAs (snoRNAs) broaden the ncRNA regulatory landscape through mechanisms of translational control, transposon silencing, and site-specific RNA modification [15].

An increasing number of studies have demonstrated the anticancer effects of natural products by regulating ncRNA expression and function in various cancers [16,17,18,19,20]. Among polyphenols, curcumin induces NRF2-dependent transactivation of the miR-34a/b/c cluster to suppress colorectal cancer metastasis through senescence and apoptosis by activating the ROS/KEAP1/NRF2 cascade [16]. Baicalein upregulates lncRNA-NEF to inactivate Wnt/β-catenin signalling through a negative feedback loop, thereby inhibiting osteosarcoma proliferation and metastasis [18]. Additionally, in the context of alkaloids, the isoquinoline alkaloid berberine modulates autophagy and glycolysis in ovarian cancer by disrupting the LINC01123/P65 interaction, decreasing P65 stability and downstream MAPK10 signalling [20]. Together, these results highlight the therapeutic potential of natural products to target ncRNA networks in cancer therapy.

In this review, we aim to (i) systematically summarize the biogenesis and functions of major ncRNA classes in cancer; (ii) integrate the current evidence on natural product–ncRNA regulation across malignant phenotypes; and (iii) evaluate the translational potential and limitations of ncRNA-based biomarkers and co-delivery strategies in natural product anticancer therapy. A detailed introduction to the biogenesis, classification, and molecular functions of major ncRNA classes is first discussed. The diverse mechanisms by which ncRNAs regulate key hallmarks of tumor progression are then summarized, with an emphasis on their oncogenic and tumor-suppressive roles. Subsequently, the modulation of these ncRNA networks by natural products and their effects on multiple malignant phenotypes, including cell proliferation, programmed cell death (PCD), metastasis, tumor microenvironment (TME) remodeling, and drug resistance, are systematically reviewed. The potential of ncRNAs as diagnostic and prognostic biomarkers in the context of natural product-based therapy and emerging targeted delivery strategies is further discussed. Finally, current challenges and future perspectives for the clinical translation of natural products targeting ncRNA networks in cancer treatment are addressed.

### Literature Search Strategy

A comprehensive literature search was conducted in PubMed from July 2021 to July 2026, using combinations of the following keywords: (“natural product*” OR “phytochemical*” OR “plant-derived compound*”) AND (“non-coding RNA*” OR “microRNA*” OR “miRNA*” OR “long non-coding RNA*” OR “lncRNA*” OR “circular RNA*” OR “circRNA*” OR “tRNA-derived fragment*” OR “piRNA*” OR “snoRNA*”) AND (“cancer” OR “tumor” OR “carcinoma” OR “malignance*”). Original research articles demonstrating that natural products regulate ncRNA expression or function in cancer models were included; reviews were used for background information. Conference abstracts, non-English publications, and studies without mechanistic data were excluded. Titles and abstracts were screened independently by two authors, and full texts were assessed against the inclusion criteria. A total of 34 articles were ultimately included.

## 2. Biological Functions and Classification of Non-Coding RNA

**Figure 1:**
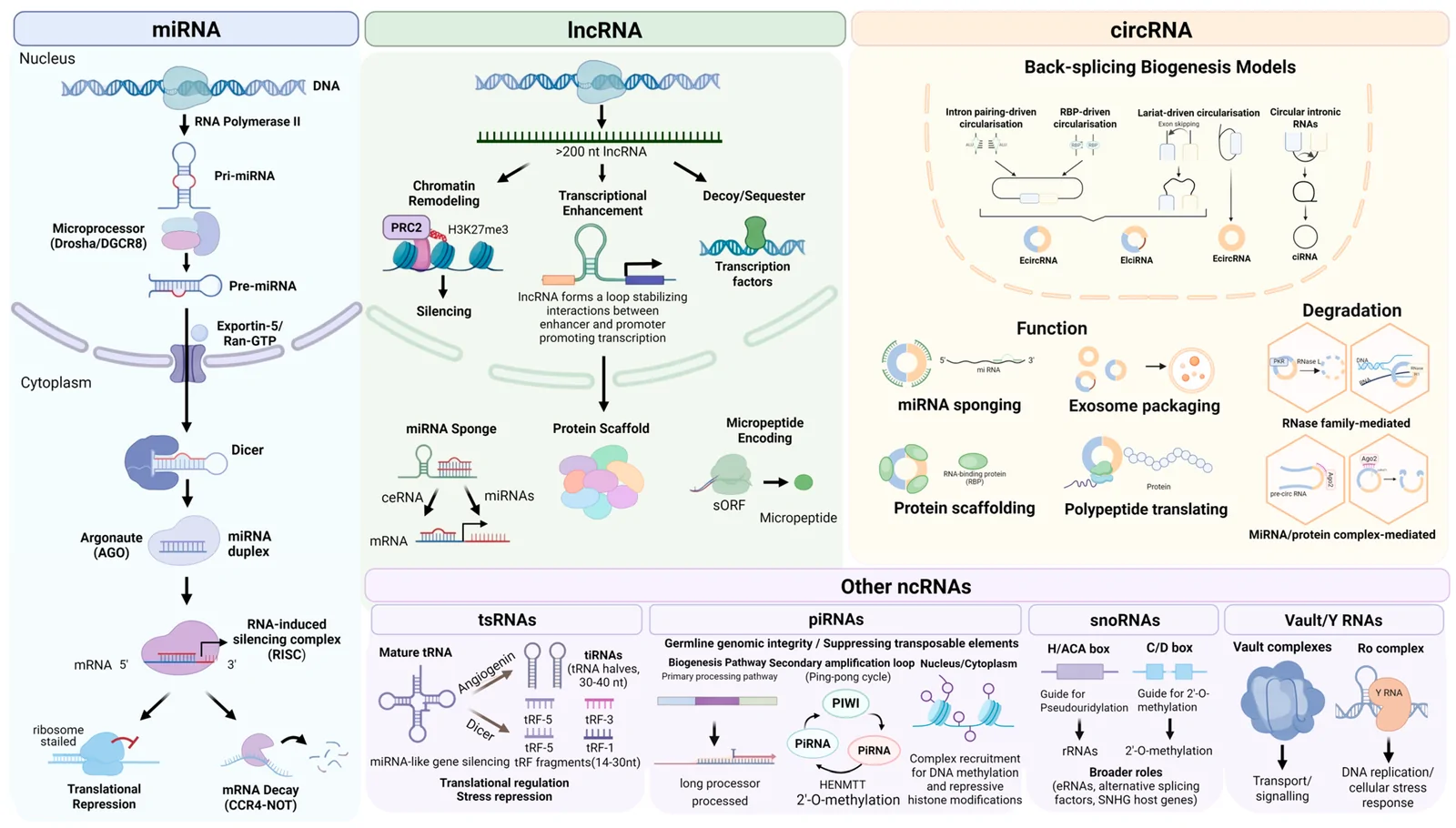
Main classification and biological functions of ncRNAs. Schematic overview of the major ncRNA classes, their biogenesis pathways, and molecular functions. The left panel depicts miRNA processing from Pol II transcription through Drosha/DGCR8 and Dicer cleavage to RISC-mediated target repression. The central panel summarizes lncRNA functions, including chromatin remodeling, transcriptional enhancement, decoy activity, miRNA sponging, protein scaffolding, and micropeptide encoding. The right panel illustrates circRNA biogenesis via intron-pairing, RBP-mediated, and lariat-driven models, with mechanisms including miRNA sponging, RBP decoys, protein scaffolding, R-loop formation, rolling-circle translation, and m6A-dependent regulation. Other classes, namely tsRNAs, piRNAs, snoRNAs, and vault/Y RNAs, are summarized at the bottom for transposon silencing, rRNA modification, and stress responses. Created in BioRender. You, Q. (2026) https://BioRender.com/jloa4tc.

NcRNAs encompass a diverse array of regulatory RNA species that govern gene expression at transcriptional, post-transcriptional, and epigenetic levels. This section systematically introduces the biogenesis pathways, structural features, and molecular mechanisms of major ncRNA classes, including miRNAs, lncRNAs, circRNAs, and other emerging ncRNAs (Figure 1 and Table 1). This review focuses on endogenously expressed non-coding RNAs. It should be noted that most of these small RNA species act within the framework of RNA interference (RNAi), a conserved post-transcriptional gene-silencing pathway that can be triggered either by endogenously expressed ncRNAs (e.g., miRNAs) or by exogenously designed small interfering RNAs (siRNAs) and short hairpin RNAs (shRNAs), which are processed by the same RISC machinery [21]. Accordingly, synthetic RNAi effectors (siRNAs, shRNAs, antagomiRs, and miRNA mimics) are discussed in this review as therapeutic tools in the context of engineered ncRNA-targeting strategies (Section 3.5 and Section 5.2).

### 2.1. MicroRNA

#### 2.1.1. Biogenesis and Processing

MicroRNAs (miRNAs) are small non-coding RNAs of approximately 19–25 nucleotides that function as master regulators of gene expression at the post-transcriptional level. miRNA biogenesis is a well-orchestrated multi-step pathway, beginning in the nucleus and ending in the cytoplasm [9].

The canonical miRNA biogenesis pathway starts with RNA polymerase II transcription, resulting in primary miRNA transcripts (pri-miRNAs) with local stem-loop structures. Inside the nucleus, the Microprocessor complex, consisting of the RNase III enzyme Drosha and its cofactor DGCR8, identifies and cuts the stem-loop junction of the pri-miRNA, producing a precursor miRNA (pre-miRNA) of around 60–70 nucleotides with a typical 2-nucleotide 3′ overhang [22]. Importantly, an alternative route of biogenesis involves a subset of miRNAs, called mirtrons, that are generated from intronic regions by the splicing machinery and bypass Drosha processing.

Following nuclear processing, pre-miRNAs are transported to the cytoplasm via Exportin-5 in a Ran-GTP-dependent manner. Once in the cytoplasm, the RNase III enzyme Dicer recognizes the pre-miRNA terminal loop and cleaves it to generate a mature miRNA duplex of approximately 22 base pairs. The duplex is subsequently loaded onto the Argonaute (AGO) protein, where strand selection happens depending on thermodynamic stability: the guide strand with lower 5′ stability is retained in the RISC, while the passenger strand is normally degraded [10]. Beyond this canonical pathway, emerging evidence has revealed alternative miRNA biogenesis mechanisms, including Dicer-independent pathways such as those involving miR-451, which utilizes AGO2 catalytic activity for maturation, and AGO-independent pathways that broaden the regulatory scope of the small RNA repertoire [23,24].

#### 2.1.2. Gene Silencing Mechanisms

The primary mechanism of miRNA gene regulation is sequence-specific binding of miRNAs to target mRNAs, primarily via seed region complementarity. The mature miRNA directs the RISC to complementary sequences, generally found in the 3′ untranslated region (UTR) of the target mRNAs. The seed region, which corresponds to nucleotides 2–8 of the miRNA sequence, is the crucial factor in target recognition [9,25].

MiRNA-mediated gene silencing occurs via two major mechanisms, translational repression and mRNA destabilisation. Translational inhibition can occur at initiation or post-initiation steps, where the RISC complex interferes with cap recognition, ribosome recruitment, or ribosome elongation [26]. Moreover, miRNAs facilitate mRNA decay through the recruitment of the CCR4-NOT deadenylase complex, resulting in the shortening of the poly(A) tail, decapping, and subsequent 5′-to-3′ exonucleolytic decay [27,28]. GW182 family proteins are crucial adaptors linking AGO proteins to the deadenylation machinery, thereby orchestrating the silencing response [29].

In addition to the canonical targeting of 3′ UTRs, miRNAs have been shown to associate with other mRNA regions such as 5′ UTRs, coding sequences, and promoter regions, thus expanding the repertoire of miRNA-mediated regulation. Moreover, recent studies have shown that miRNAs are also capable of functioning in the nucleus to modulate gene expression at the transcriptional level, providing an additional level of complexity to their regulatory potential [30]. Imperfect pairing in animals results mainly in translational inhibition and mRNA destabilisation [8,31].

### 2.2. Long Non-Coding RNA

#### 2.2.1. Genomic and Phylogenomic Features

Long non-coding RNAs are defined as RNA transcripts that exceed 200 nucleotides and lack or have limited protein-coding potential. The lncRNA landscape is remarkably diverse in terms of genomic origin, structural complexity, and subcellular localisation [32].

LncRNAs are categorised into several types based on their genomic location relative to protein-coding genes: (i) intergenic lncRNAs (lincRNAs) are transcribed from the genomic regions located between protein-coding genes; (ii) antisense lncRNAs are transcribed from the opposite strand of coding genes and can overlap with their sense counterparts; (iii) intronic lncRNAs are transcribed entirely within the intronic regions; (iv) sense or antisense overlapping lncRNAs share genomic loci with coding transcripts; (v) enhancer RNAs (eRNAs) are transcribed from enhancer regions and are transcriptionally activated [10]. Moreover, many lncRNAs originate from single-exon precursors, such as MALAT1 and NEAT1, and are localised in specific nuclear compartments [32].

LncRNAs are transcribed by RNA polymerase II and are co-transcriptionally processed like mRNAs, including 5′ capping, splicing, and polyadenylation. However, lncRNAs generally contain fewer exons, are less abundant, and their expression pattern exhibits higher tissue- and cell-type-specificity [10]. A further unique characteristic of lncRNAs is their dynamic subcellular localisation, which is closely related to their functional mechanisms: nuclear lncRNAs are often involved in chromatin regulation and transcriptional control, whereas cytoplasmic lncRNAs are more frequently related to post-transcriptional regulation, including modulation of mRNA stability, translation control, and signalling pathway modulation [8]. Significantly, lncRNAs exhibit a wide range of stabilities, with short-lived lncRNAs involved in rapid regulatory responses and long-lived lncRNAs involved in sustained architectural or scaffolding roles [33].

#### 2.2.2. Molecular Mechanisms of Action

Reflecting the structural complexity and subcellular plasticity of lncRNAs, the molecular mechanisms by which they exert their regulatory actions are staggering. The functional repertoire of lncRNAs can be broadly divided into a number of distinct modes of action [34].

At the level of chromatin, lncRNAs can act as molecular scaffolds that recruit chromatin-modifying complexes to specific genomic loci. For example, lncRNAs can recruit polycomb repressive complex 2 (PRC2) to target genes to facilitate the deposition of H3K27me3 and transcriptional silencing [35,36]. lncRNAs can sequester transcription factors or regulatory proteins away from their target sites and thereby modulate transcriptional output without direct interaction with chromatin. In addition, some lncRNAs have been shown to function as transcriptional enhancers by forming RNA-DNA triplex structures or stabilising enhancer–promoter looping, thus promoting long-range chromatin interactions [10] structures or stabilising enhancer.

At the post-transcriptional level, several mechanisms have been reported by which lncRNAs control mRNA processing, stability and translation. LncRNAs can change the alternative splicing patterns by sequestering splicing factors or base-pairing with pre-mRNA to cover splice sites [32]. Many lncRNAs in the cytoplasm function as competing endogenous RNAs (ceRNAs) or miRNA sponges, thereby competitively inhibiting miRNA-mediated repression. Additionally, lncRNAs can regulate mRNA stability by direct base-pairing or by recruiting RNA-binding proteins that stabilise or destabilise target transcripts [10,37].

Another layer of function of lncRNAs is in the regulation of protein localisation and activity. LncRNAs can function as molecular scaffolds bringing proteins into close proximity to enable complex assembly, or as decoys to sequester proteins away from sites of action. Some lncRNAs modulate post-translational modifications of proteins, such as phosphorylation, ubiquitination, and acetylation, to indirectly modulate the activity of signalling cascades [10,38]. In addition, recent evidence has shown that some lncRNAs harbour short open reading frames (sORFs) that have the capacity to encode functional micropeptides, challenging the traditional dichotomy between coding and non-coding transcripts [39]. These lncRNA-encoded peptides have been shown to be involved in a variety of biological processes, adding another dimension to the functional complexity of the lncRNA landscape.

### 2.3. Circular RNA

#### 2.3.1. Biogenesis and Degradation

CircRNAs represent a distinct class of ncRNAs characterised by a covalently closed loop structure, formed through the process of back-splicing, where a downstream 5′ splice site is connected to an upstream 3′ splice site. The unique circular structure of circRNAs provides them with remarkable stability and resistance to degradation by exonucleases [13].

The biogenesis of circRNAs primarily occurs through back-splicing, a non-canonical splicing event competing with linear pre-mRNA splicing. Three major models have been proposed: intron-pairing-driven circularisation, in which complementary sequences (especially Alu repeats) in flanking introns bring splice sites into proximity; RBP-mediated circularisation, in which RNA-binding proteins dimerise to link flanking introns [40]; and lariat-driven circularisation, in which skipped exons form lariat structures that are internally back-spliced [41]. The competitive interaction between back-splicing and canonical linear splicing determines the relative abundance of circRNAs versus their linear counterparts [42].

In addition to well-characterized cytoplasmic circRNAs derived from coding exons, multiple distinct subtypes have been identified: exon-intron circRNAs (EIciRNAs), which retain intronic sequences and regulate parental gene transcription in the nucleus; intronic circRNAs (ciRNAs), generated from non-debranched intronic lariats; and mitochondrial genome-derived circRNAs (mecciRNAs) [43,44,45].

The degradation of circRNAs is also tightly regulated: DIS3 mediates global circRNA decay by recognising U-rich motifs [46]; RNase L cleaves circRNAs upon innate immune activation; miRNA/Argonaute-mediated cleavage (e.g., miR-671-induced CDR1as cleavage) and m^6^A modification via the YTHDF2 pathway further contribute to circRNA turnover [47]; and structure-mediated RNA decay by G3BP1 and UPF1 provides an additional surveillance layer for highly structured circRNAs [48].

#### 2.3.2. Molecular Functions

CircRNAs perform a broad range of molecular functions through interactions with various biomolecules, including miRNAs, proteins, mRNAs, and DNA. The functional properties of circRNAs make them versatile regulators of gene expression and cellular signalling [49].

The most well-characterized role of circRNAs is their capacity to act as miRNA sponges. CircRNAs frequently harbour multiple miRNA response elements (MREs) and may act as competitive miRNA sponges, derepressing miRNA target genes. CDR1as/ciRS-7 is the prototype of circRNA-mediated miRNA sponging, with >70 conserved miR-7 binding sites, and acts as a potent negative regulator of miR-7 activity [50]. Although many studies have been done, the rigorous validation of functional circRNA-miRNA interactions needs to be experimentally confirmed by bioluminescence reporter assays, AGO2 crosslinking and immunoprecipitation, and functional rescue experiments, rather than computational prediction alone.

Besides miRNA sponging, circRNAs dynamically interact with proteins through multiple mechanisms. CircRNAs can act as a protein sponge by binding to and sequestering certain proteins from their normal sites of action. They can also function as protein scaffolds, bringing several protein partners into proximity for complex assembly and activation of signalling cascades. CircRNAs can act as a protein decoy, competing with the natural protein binding sites and redirecting proteins from their physiological targets. Also, circRNAs can act as recruiters and direct proteins to particular subcellular locations or modulate their enzymatic activity [40].

Besides, circRNAs participate in transcriptional regulation via several mechanisms. Nuclear-localized EIciRNAs can bind to small nuclear ribonucleoprotein U1 (snRNP U1) and RNA polymerase II to promote the transcription of their parent genes. Additionally, circRNAs can interfere with pre-mRNA splicing by binding to and sequestering splicing factors, thus influencing the balance between circular and linear transcript production [51]. The competition between circRNA biogenesis and canonical linear splicing was first demonstrated with the splicing factor Muscleblind (MBL), which promotes its own circularisation at the expense of linear transcript production, establishing a negative feedback regulatory loop.

Recent research has revealed more functional aspects of circRNAs. Recent evidence shows that some circRNAs can interact directly with DNA to form circRNA-DNA hybrid structures, called circR-loops, which are involved in regulating alternative splicing, chromatin remodelling, and genomic instability [52,53,54,55]. Some circRNAs can also interact with mRNA molecules directly and control their stability and translation via miRNA activity-independent mechanisms [56,57,58].

Furthermore, certain circRNAs containing open reading frames (ORFs) can serve as templates for translation into proteins. In contrast to canonical cap-dependent translation, circRNA translation is mediated by cap-independent mechanisms, mainly involving internal ribosome entry sites (IRESs) or m6A-mediated initiation [59]. M^6^A and IRES elements recruit the 40S ribosomal subunit in a cap-independent manner. This change recruits reader proteins, such as YTHDF3, and initiation factors, such as eIF4G2 [60]. Some circRNAs can be translated through rolling-circle translation, producing protein products, while others encode functional peptides or proteins that participate in various biological processes [61]. Although the field of circRNA translation has advanced considerably [62,63,64], technical challenges remain in the detection of low-abundance peptides produced by circRNA translation and in dissecting molecular details of internal ribosome recruitment [65,66].

Finally, circRNAs participate in intercellular communication through their incorporation into extracellular vesicles (EVs), especially exosomes. CircRNAs are enriched and stably retained in exosomes, suggesting a selective sorting mechanism [50]. The mechanisms involved in circRNA loading into exosomes include RBP-mediated packaging, m^6^A modification, allowed sorting, and stress-induced modulation of exosomal cargo composition. These exosomal circRNAs can be absorbed by recipient cells, where they alter cellular processes and gene expression, increasing the intricacy of intercellular regulation. Collectively, these diverse functions position circRNAs as versatile mechanistic nodes that connect multiple signalling pathways [67,68,69].

### 2.4. Other Non-Coding RNAs

#### 2.4.1. tRNA-Derived Fragments

Transfer RNA-derived fragments (tsRNAs) represent a novel class of small ncRNAs generated by site-specific cleavage of mature tRNAs or tRNA precursors. Once viewed merely as byproducts of tRNA turnover, tsRNAs are now recognised as functionally versatile molecules with specific biogenesis and regulatory mechanisms [70].

TsRNAs are generally classified into two main categories according to their origin and cleavage sites. Under stress conditions, the ribonuclease angiogenin cleaves in the anticodon loop, releasing tRNA halves (tiRNAs) of about 30–40 nucleotides, including 5′-tiRNAs and 3′-tiRNAs. In contrast, tRNA-derived small RNA fragments (tRFs) are short molecules of 14–30 nucleotides produced by Dicer or other nucleases at specific sites like tRF-5 (derived from the 5′ end), tRF-3 (derived from the 3′ end), and tRF-1 (derived from the 3′ trailer sequence of precursor tRNAs) [71]. TsRNA biogenesis is dynamically regulated by cellular stress, nutrient availability and epigenetic modifications, with specific cleavage patterns being associated with distinct physiological conditions.

TsRNAs exert their effects via various molecular mechanisms. Some tsRNAs can form complexes with Argonaute to mediate post-transcriptional gene silencing in a miRNA-like manner by recognising complementary sequences in target mRNAs [70]. Furthermore, tsRNAs have been reported to regulate translation by competing with translation initiation factors for binding to mRNAs, forming RNA–protein complexes that interfere with ribosome assembly, or by globally repressing protein synthesis under stress conditions [72]. Furthermore, tsRNAs may regulate retrotransposon activity, modulate RNA processing, and influence epigenetic inheritance, reflecting their diverse regulatory repertoire beyond translational control. The functional specificity of tsRNAs is dictated by their nucleotide sequence, modification status, and association with specific RNA-binding proteins.

#### 2.4.2. PIWI-Interacting RNAs

Another set of small ncRNAs is represented by PIWI-interacting RNAs (piRNAs). These small RNAs are 24–31 nucleotides long and bind exclusively to PIWI proteins, a subgroup of the AGO protein family. PiRNAs were first identified in the germline cells and are best known for their role in preserving the integrity of the genome by suppressing the transposable elements [73,74,75].

There are two major signalling pathways of piRNA biogenesis: the primary processing pathway and the secondary amplification loop (ping-pong cycle). In the context of primary processing, piRNA clusters are transcribed into long single-stranded precursors that are exported to the cytoplasm and cleaved by endonucleases, including PLD6/MITOPLD and Zucchini, to generate piRNA intermediates that are loaded onto PIWI proteins. The 3′ ends of piRNA intermediates are then trimmed by exonucleases and subsequently modified by the 2′-O-methyltransferase HENMT1, a modification that provides increased stability and protection from degradation [76]. The ping-pong cycle is a feed-forward mechanism that amplifies the production of piRNAs. Primary piRNAs use PIWI proteins to cleave complementary transposon transcripts, which produce secondary piRNAs. Secondary piRNAs associate with other PIWI proteins to produce more primary-like piRNAs, thus establishing an amplification loop.piRNAs. Secondary piRNAs associate with other PIWI proteins to produce more primary-like piRNAs, thus establishing an amplification loop.

PiRNAs mainly exert their functions through transcriptional and post-transcriptional repression of transposable elements. In the nucleus, PIWI-piRNA complexes identify nascent transposon transcripts by sequence complementarity and recruit chromatin-modifying enzymes that guide DNA methylation and repressive histone modifications to transposon sites. In the cytoplasm, piRNAs can mediate post-transcriptional cleavage of transposon mRNAs by the slicer activity of PIWI proteins. In addition to transposon silencing, piRNAs have been implicated in the regulation of protein-coding genes, mRNA stability and translation, suggesting broader biological functions beyond genome defence [77,78].

#### 2.4.3. Small Nucleolar RNAs and Other ncRNAs

Small nucleolar RNAs (snoRNAs) are a class of evolutionarily conserved ncRNAs with a size of 60–300 nucleotides that play a major role in the chemical modifications of ribosomal RNAs (rRNAs) and other RNA substrates. According to the conserved sequence motifs and structural features, snoRNAs are divided into two major groups: H/ACA box snoRNAs that direct pseudouridylation of target RNAs and C/D box snoRNAs that guide 2′-O-methylation [79].

Beyond their canonical roles in rRNA modification, accumulating evidence has indicated that snoRNAs and their derivatives, such as snoRNA-derived RNAs (sdRNAs), have broader regulatory functions. SnoRNAs have been demonstrated to regulate pre-mRNA alternative splicing by sequestering splicing factors, modulate mRNA editing, and influence the processing of other ncRNAs. The regulatory landscape is further complicated by the fact that many snoRNA host genes, particularly those of the SNHG (small nucleolar RNA host gene) family, not only give rise to snoRNAs but also produce longer transcripts that act as lncRNAs [80].

Besides the well-characterized ncRNA classes mentioned above, other classes of ncRNAs contribute to the regulatory repertoire of the cell. Vault RNAs are derived from the vault ribonucleoprotein complex and have been implicated in cellular transport and signalling [81]. Y RNAs are components of the Ro ribonucleoprotein complex involved in DNA replication initiation and cellular stress responses. These additional species, while less studied than the major classes of ncRNAs, add to the functional diversity of the non-coding transcriptome and continue to reveal novel regulatory mechanisms [82]. Further studies of these understudied ncRNAs are expected to expose more layers of gene regulatory complexity and expand the ncRNA functional landscape.

## 3. Regulatory Functions of Non-Coding RNAs in Tumor Progression

**Figure 2:**
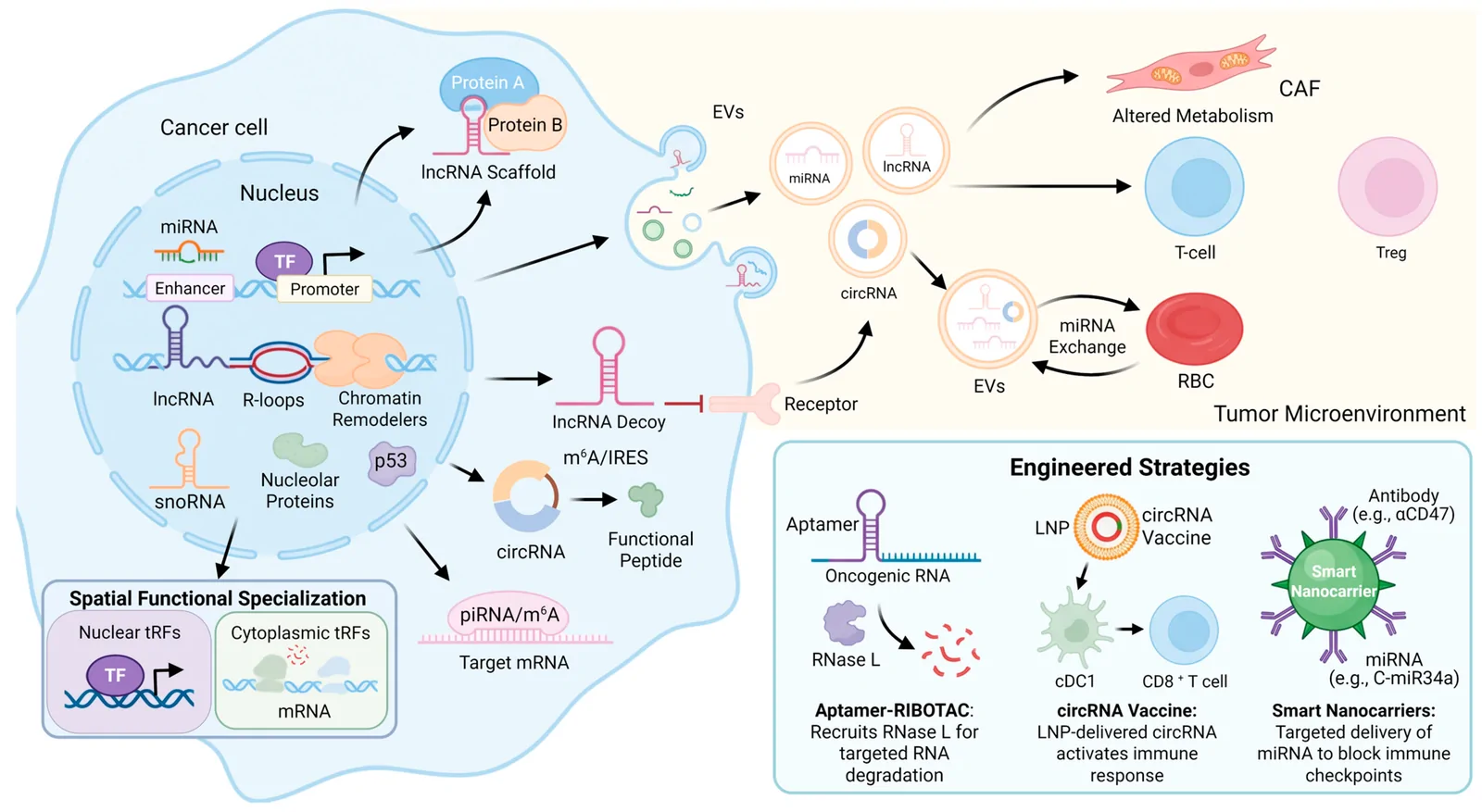
Regulatory functions of ncRNAs in tumor progression and engineered therapeutics. Within cancer cells, lncRNAs function as scaffolds, decoys, and chromatin remodelers, while circRNAs, snoRNAs, and piRNA-m6A programs converge on p53 signaling and translational control. ncRNAs are packaged into extracellular vesicles and exchanged with cancer-associated fibroblasts, T cells, regulatory T cells, and erythrocytes, reprogramming stromal metabolism and immunosuppression. These dependencies are exploited by engineered platforms: aptamer-RIBOTACs degrade oncogenic transcripts, cDC1-targeted lipid nanoparticle circRNA vaccines elicit CD8^+^ T-cell responses, and antibody-decorated smart nanocarriers deliver miRNA mimics such as C-miR34a to bypass immune checkpoints, delineating a translational pipeline from ncRNA biology to RNA-centric cancer therapy. Created in BioRender. You, Q. (2026) https://BioRender.com/hrhhyr4.

The studies summarized in this section are based on heterogeneous experimental systems, ranging from cancer cell lines and organoids to xenograft and patient-derived models, with a minority incorporating clinical samples. Evidence derived from cell-based assays and xenograft models should therefore be interpreted as mechanistic and preclinical, whereas conclusions supported by patient-derived or clinical data are indicated where applicable. Given the context-dependent and sometimes opposing functions of individual ncRNAs, broad conclusions based on isolated studies should be drawn with caution (Figure 2).

### 3.1. MicroRNAs in Tumorigenesis

MicroRNAs orchestrate tumor development through their dual roles as oncogenes (oncomiRs) and tumor suppressors. By targeting oncogenes, tumor suppressor genes, and core signaling pathways (e.g., PI3K/AKT, Wnt/β-catenin, p53), miRNAs regulate virtually all hallmarks of cancer, including sustained proliferation, resistance to cell death, invasion and metastasis, metabolic reprogramming, immune evasion, and therapeutic resistance [8,9]. Notably, the same miRNA can exert opposing functions in different tumor types or cellular contexts, reflecting the complexity of miRNA–target networks. Below, we highlight representative examples of miRNA dysregulation in tumorigenesis, with emphasis on non-canonical nuclear functions and intercellular communication through extracellular vesicles.

#### 3.1.1. Nuclear Non-Canonical Functions of miRNAs

Further research has shown that miRNA regulation is not restricted to canonical 3′UTR targeting but also involves direct chromatin-associated functions in the nucleus. In hepatocellular carcinoma (HCC), Chen et al. showed that miR-483-5p directly targets enhancer regions of IGF2 and H19, promoting enhancer–promoter chromatin looping by eRNA-MED1 complexes to upregulate these oncogenes [83]. miR-107 was identified by Castoldi et al. as a miRNA consistently downregulated in HCC, targeting KIF23 to regulate cytokinesis, and the overexpression or silencing of Kif23 significantly inhibits c-Myc-NRAS-induced hepatocarcinogenesis [84]. In colorectal cancer (CRC), nuclear miR-494-5p recruits AGO proteins to the KAT2B enhancer to change local histone modifications and disrupt MED1-p300-RNAP II co-activator assembly and enhancer–promoter looping, leading to suppression of the c-JUN-driven pro-metastatic program [85].NRAS-induced hepatocarcinogenesis [85]. In colorectal cancer (CRC), nuclear miR-494-5p recruits AGO proteins to the KAT2B enhancer to change local histone modifications and disrupt MED1-p300-RNAP II co-activator assembly and enhancer—promoter looping, leading to suppression of the c-JUN-driven pro-metastatic program.

#### 3.1.2. EV-Mediated Intercellular Communication of miRNAs

In addition to these cell-autonomous nuclear functions, miRNAs also act via EV-mediated intercellular communication. In ovarian cancer, Zheng et al. showed that cisplatin selectively enhances EV secretion from cancer cells and increases the enrichment of these EVs with miR-181a-5p, which promotes CD4^+^ T cell differentiation toward immunosuppressive regulatory T cells (Tregs) via SIRT1 suppression and FOXP3 derepression [86]. In lung cancer, Li et al. identified a bidirectional exchange loop where exosomal miR-93-5p is transferred from RBCs to tumour cells to inhibit PTEN, and tumour cells transfer miR-93-5p to RBCs. Therapeutic inhibition of miR-93-5p by antisense oligonucleotides (ASO) suppresses tumour progression and prolongs survival in murine models [87]. In addition to these tissue-specific examples, Hu et al. found a more general mechanism whereby cancer cells, under glucose starvation and low iron, specifically release miR-33a via EVs with the assistance of aconitase 1 (ACO1), which interacts with miR-33a for EV loading. This EV-miR-33a inhibits putrescine synthesis in CAFs and decreases stress granule formation via the KDM5C/H3K4me3/TIA1 axis [88].

### 3.2. LncRNAs in Malignant Tumors

#### 3.2.1. ceRNA Networks

Competing endogenous RNA (ceRNA) networks are an essential mechanism of lncRNA function, in which lncRNAs sponge miRNAs and thereby derepress miRNA target mRNAs. Wu et al. found that CAFs-secreted exosomal lncRNA PRKCQ-AS1 sponges miR-200a-3p in ER+ breast cancer cells, resulting in the upregulation of MKP1, inhibition of MAPK/JNK signalling pathways, and inhibition of tamoxifen-induced apoptosis. The clinical significance of this ceRNA axis was verified in a clinical cohort of 471 patients, where a significant association was found between PRKCQ-AS1 activation levels and endocrine therapy outcome [89]. These findings extend the conventional view of ceRNA networks beyond intracellular circuits to intercellular regulation, highlighting the importance of stromal–tumor crosstalk in ncRNA-mediated drug resistance.

#### 3.2.2. Epigenetic and Transcriptional Regulation

LncRNAs have various epigenetic and transcriptional regulatory functions such as reprogramming of splicing, chromatin reorganisation, and transcriptional feedback. In HCC, RAB30-DT is a CREB1-activated lncRNA that directly binds to the splicing kinase SRPK1 and promotes its nuclear translocation, as reported by Si et al. This remodels the alternative splicing landscape of cell-cycle regulators, including CDCA7, which drives tumour stemness and malignant progression [90]. Moreover, Zhou et al. reported an NFYB-LINC01137 positive feedback loop in which stabilised NFYB induces the expression of CCL20, CXCL2 and IL-1β to promote M2 macrophage polarization in HCC progression. lncRNA-mediated epigenetic regulation is incorporated into the TME remodelling, indicating that blocking this loop may impact both cancer cell-intrinsic and extrinsic drivers of HCC progression simultaneously [91]. In the context of CRC liver metastasis, Lv et al. showed that STEAP3-AS1 creates R-loops with its parental gene STEAP3 to control the chromatin remodeler BRG1, changing MMP9 chromatin accessibility through the BRG1/ERG/P300 complex, thus promoting CRC liver metastasis [92]. In multiple myeloma, a CRISPR-Cas13d functional transcriptomic screen identified a specific variant of SNHG6 that localised to the endoplasmic reticulum and interacted with heat-shock proteins to maintain proteostasis [93]. Individual isoforms, rather than full gene loci, represent the functional units of lncRNA, implying that the next generation of lncRNA functional genomics will demand isoform-resolved loss-of-function approaches.SNHG6 that localised to the endoplasmic reticulum and interacted with heat-shock proteins to maintain proteostasis [94]. Individual isoforms, rather than full gene loci, represent the functional units of lncRNA, implying that the next generation of lncRNA functional genomics will demand isoform-resolved loss-of-function approaches.

#### 3.2.3. Scaffold and Decoy Functions

LncRNAs may act as molecular scaffolds to either promote or disrupt signalling complexes or as decoys that competitively sequester proteins away from their canonical binding partners. Yang et al. showed that AR-regulated lncRNA PRCAT71 serves as a scaffold to recruit RNA-binding protein KHSRP to bind and stabilise AR mRNA, thereby establishing an AR-PRCAT71 positive-feedback loop that is critical for sustained AR signalling [94]. The dual role of metastasis suppression and immune activation in one lncRNA scaffold suggests that some lncRNAs may function as ‘pleiotropic regulatory hubs’ that simultaneously regulate multiple cancer hallmarks.

In oral cancer, Chen et al. discovered that HIF-1α-induced lncRNA EUDAL functions as a decoy by competing with c-Cbl and Grb2 to bind to epidermal growth factor receptor (EGFR). This competition causes sustained EGFR phosphorylation of downstream signalling, leading to chemotherapy resistance via the STAT3/BNIP3 autophagy pathway [95]. In gastric cancer (GC), Tang et al. found that the EGR1-transactivated lncRNA TENM3-AS1 directly interacts with and deubiquitinates the RNA-binding protein hnRNPK to stabilise it, increasing the stability of FASN mRNA and reprogramming fatty acid metabolism to promote metastasis [96]. In Triple-Negative Breast Cancer (TNBC), Elango et al. conducted a CRISPR-Cas9 screen and identified TPL1 (TNBC Promoting LncRNA 1), which regulates ECM–receptor interaction and PI3K-Akt signalling [97]. In CRC, Chen et al. identified lncRNA 60967.1 as a downregulated tumour suppressor, whose restoration increases levels of all-trans retinoic acid, enhances IFN-γ-induced apoptosis, and synergises with anti-PD-1 therapy [98]. Moreover, CRCMSL was reported to induce ACC1 phosphorylation to restrict monounsaturated fatty acid synthesis, reducing membrane fluidity and sensitising CRC cells to ferroptosis [99]. Together, lncRNAs are considered to be uniquely versatile regulatory hubs able to integrate multiple regulatory functions, scaffolding, decoy activity, and transcriptional control initially into a single molecule.

### 3.3. CircRNA in Cancer Development

#### 3.3.1. miRNA Sponges

The miRNA sponge function of circRNAs remains the most extensively characterized mechanism. In HCC, Zhang et al. identified circTTC13 as a significantly upregulated circRNA that sponges miR-513a-5p to upregulate SLC7A11, suppressing ferroptosis and driving sorafenib resistance [100]. In contrast, Yu et al. demonstrated that circSMARCA5 (hsa_circ_0001445) functions as an HCC-suppressive sponge, simultaneously sequestering miR-17-3p and miR-181b-5p to upregulate the tumour suppressor TIMP3, with systemic delivery of circSMARCA5 suppressing tumour growth and metastasis in vivo [101].

#### 3.3.2. RBP Regulators

Besides miRNA sponging, circRNAs also regulate their functions via interaction with RBPs. In CRC, Dong et al. reported an EIF4A3/circPTGR1/miR-4725-5p positive-feedback loop to upregulate the FAK/AKT pathway, with functional validation in patient-derived CRC organoids [102]. Moreover, Hu et al. found that circPHLPP2 interacts with the RBP ILF3 and enhances its nuclear localisation to increase IL36γ transcription, hindering NK cell infiltration and contributing to anti-PD-1 resistance in CRC [103]. In addition, Lin et al. characterized circTATDN3 as a dual-function molecule in CRC: it serves as an adaptor enhancing LDHA–FGFR1 interaction to promote the Warburg effect, while simultaneously sequestering miR-511-5p to upregulate LDHA expression [104]. In nasopharyngeal carcinoma, Peng et al. discovered that circCLASP2 acts as a molecular scaffold to recruit DHX9 to unravel RNA G-quadruplex (rG4) structures close to the translation initiation site of PCMT1 mRNA, thus facilitating PCMT1 translation [105]. In head and neck squamous cell carcinoma (HNSCC), Wu et al. reported that the m6A-modified circHECTD2 maintains SMAD2 expression through ceRNA mechanisms [106]. In TNBC, Wang et al. revealed that LDHA Y10 phosphorylation drives EV-circSEPT9 secretion, which upregulates KIAA1429 via ceRNA and enhances m6A modification to promote doxorubicin resistance [107]. In non-small cell lung cancer (NSCLC), Chen et al. observed that downregulated hsa_circ_0050386 interacted with SRSF3 to suppress FN1 expression, thus hindering NSCLC progression [108]. The ceRNA, RBP scaffolding, and epitranscriptomic regulation combination of the circRNA class endows these molecules with unique versatility as post-transcriptional regulators.enhancing LDHA–FGFR1 interaction to promote the Warburg effect, while simultaneously sequestering miR-511-5p to upregulate LDHA expression [105]. In nasopharyngeal carcinoma, Peng et al. discovered that circCLASP2 acts as a molecular scaffold to recruit DHX9 to unravel RNA G-quadruplex (rG4) structures close to the translation initiation site of PCMT1 mRNA, thus facilitating PCMT1 translation.

#### 3.3.3. Translation and Functional Peptides

The finding that circRNAs can be translated into functional peptides has added a novel dimension to their regulatory repertoire. In the context of CRC liver metastasis, Zeng et al. showed that circ-YAP produces a truncated YAP-220aa protein in CRC through m6A-mediated internal ribosome entry. YAP-220aa competitively interacts with LATS1 to promote nuclear translocation and YAP dephosphorylation, leading to activation of YAP/TEAD target genes. Interestingly, the YAP/TEAD complex also promotes circ-YAP transcription itself, thus forming a self-amplifying feedforward loop to promote CRC liver metastasis [109]. This mechanism exemplifies how peptides encoded by circRNAs can amplify oncogenic signalling through positive feedback, a regulatory architecture that would be challenging to achieve with proteins derived from linear RNA. A key future direction will be to detect circRNA translation in other cancer contexts, for which ribosome profiling, mass spectrometry, and in vivo functional validation will be needed.

### 3.4. Other Non-Coding RNAs in Tumor Pathogenesis

#### 3.4.1. Small Nucleolar RNAs (snoRNAs)

SnoRNAs have emerged as non-canonical regulators in tumor pathogenesis. In HCC, SNORD17 is significantly upregulated to promote tumor progression. SNORD17 binds nucleolar proteins NPM1 and MYBBP1A, which prevents p53 nuclear translocation and transcriptional activation. This creates a positive-feedback loop of p53-SNORD17. Importantly, ASO-mediated silencing of SNORD17 notably suppresses HCC tumour growth in vivo, demonstrating the therapeutic potential of targeting snoRNAs [110]. Collectively, the findings underscore that the functions of snoRNAs in cancer extend well beyond their classical roles in RNA modification.

#### 3.4.2. PIWI-Interacting RNAs (piRNAs)

PiRNAs have emerged as versatile regulators in cancer, operating through mechanisms far beyond their canonical role in silencing transposons in the germline. In lung adenocarcinoma (LUAD), Zhan et al. identified piRNA-137463 as a driver of progression that regulates de novo cholesterol biosynthesis through a multi-component ceRNA network involving lncRNALOC100128494 and miR-24-3p, ultimately modulating INSIG1 expression. Inhibition ofpiRNA-137463 with Antago piRNA suppresses tumor growth and metastasis and enhances anti-PD-1 therapeutic efficacy in immune-competent mouse models [111]. In prostate cancer (PCa), Ben et al. discovered that piRNA PROPER employs an m6A-dependent RNA circularization mechanism to regulate DUSP1. Specifically, piRNA PROPER links the EIF2S3 at the 5′UTR with the YTHDF2/YBX3 at the 3′UTR to promote the degradation of DUSP1 mRNA, which in turn activates p38 MAPK signalling [112]. The overlap of piRNAs with ceRNA networks, m6A modification, and environmental response pathways suggests a functional versatility comparable to miRNAs, yet these interactions remain poorly understood and warrant further investigation.piRNA PROPER links the EIF2S3 at the 5′UTR with the YTHDF2/YBX3 at the 3′UTR to promote the degradation of DUSP1 mRNA, which in turn activates p38 MAPK signalling [113]. The overlap of piRNAs with ceRNA networks, m6A modification, and environmental response pathways suggests a functional versatility comparable to miRNAs, yet these interactions remain poorly understood and warrant further investigation.

#### 3.4.3. Emerging ncRNAs: tRFs, tsRNAs, and Others

Transfer RNA-derived fragments (tRFs) and tRNA-derived small RNAs (tsRNAs) are a rapidly expanding class of regulatory small ncRNAs in cancer with roles ranging from metabolic regulation and cell death control to therapy response. In HCC, Han et al. reported that tRF-Glu-i-0545 (tRF-E) promotes ferroptosis by blocking VDAC2–Nedd4 interaction to stabilize VDAC2 and activate iron-dependent lipid peroxidation [113]. In PCa, Guo et al. demonstrated that the tumor-induced molecule tRF-21 has subcellular compartment-specific functions. Nuclear tRF-21 upregulates LAP3, while cytoplasmic tRF-21 inhibits GSTM3, thereby cooperatively increasing ROS and activating AKT signaling [114]. This “spatial functional specialization” is a fundamentally new paradigm in small ncRNA biology and will require high-resolution spatial analytical approaches.

RNA modifications are required for both biogenesis and target selectivity of tRFs/tsRNAs. In bladder cancer, Ying et al. demonstrated that METTL1 generates m7G-modified tsRNA (mtiRL) to accelerate cancer progression, which promotes Y24 phosphorylation and nuclear translocation [115]. Meanwhile, Su et al. revealed that TRMT6/61A-dependent m1A modification regulates tRF seed-region activity, influencing the unfolded protein response and gene silencing to promote bladder cancer [116]. In CRC, METTL1-modified m7G tsRNA-GlyGCC targets SPIB to regulate JAK1/STAT6 signaling and promote 5-FU resistance [117], while tRF-Glu-TTC-013 drives CRC liver metastasis via the CREB3L1-AMDHD1 metabolic pathway [118]. RNA modifications (m1A, m7G, m6A) in governing tRF/tsRNA function establish an epitranscriptomic–ncRNA regulatory axis, which is expected to be further investigated in future ncRNA cancer research.

### 3.5. Engineered Strategies Targeting ncRNA Systems

Engineered strategies targeting ncRNA systems can be categorized into three complementary classes: oligonucleotide-based approaches that directly silence or restore specific ncRNAs, vaccine- and immune-based approaches that harness ncRNA antigens or conjugates to elicit antitumor immunity, and nanoparticle-based delivery systems that improve the pharmacokinetics and tumor specificity of ncRNA therapeutics.

#### 3.5.1. Oligonucleotide-Based Strategies

In broad solid tumors, Fang et al. designed an aptamer-RIBOTAC (ARIBOTAC) chimera that couples tumor-specific aptamer-mediated cell recognition with RNase L recruitment for precise oncogenic miRNA degradation, achieving higher tumor selectivity than conventional antisense oligonucleotides [119]. Supplementary nucleic acid degradation tools include miR-responsive DNA nanomachines targeting miR-21/31 for cutaneous squamous cell carcinoma, which trigger nucleic acid cleavage upon recognizing tumor-enriched miRNAs [120].

#### 3.5.2. Vaccine- and Immune-Based Approaches

For vaccine-based ncRNA therapeutic approaches, Jiang et al. developed an HCC-targeted circGPC3 vaccine combined with a TLR4 agonist. This formulation enhances cross-talk between CD8^+^ T cells and type 1 conventional dendritic cells (cDC1), displaying stronger anti-tumor immunotherapeutic effects than traditional linear mRNA vaccines [121]. For delivery systems, in the context of immunotherapy nanocarriers, Ryu et al. devised an anti-CD47 antibody–miR-34a conjugate (aCD47-C-miR34a) that blocks the CD47 immune checkpoint, combining TNBC suppression and immune activation [122].

#### 3.5.3. Nanoparticle-Based Delivery Systems

For multi-target brain tumor nano-delivery, in orthotopic glioblastoma, Saha et al. utilized brain-penetrant nanoparticles with MRI-guided focused ultrasound to deliver miR-382 and miR-340, achieving multi-target inhibition and prolonged survival [123]. Other representative intelligent nanomedicine platforms include gold nanoparticle-based photothermal-immunotherapy systems for general solid tumors, nanocomposite hydrogels to modulate regulatory T cells within the TME, and nanoparticle carriers loaded with MYC siRNA or miR-218 to normalize abnormal tumor blood vessels and boost combined chemoimmunotherapy efficacy [124,125,126]. Collectively, these platforms underscore the growing translational promise of ncRNA-targeted therapies.

## 4. Multiple Malignant Phenotypes Regulated by Natural Products via Non-Coding RNAs

**Figure 3:**
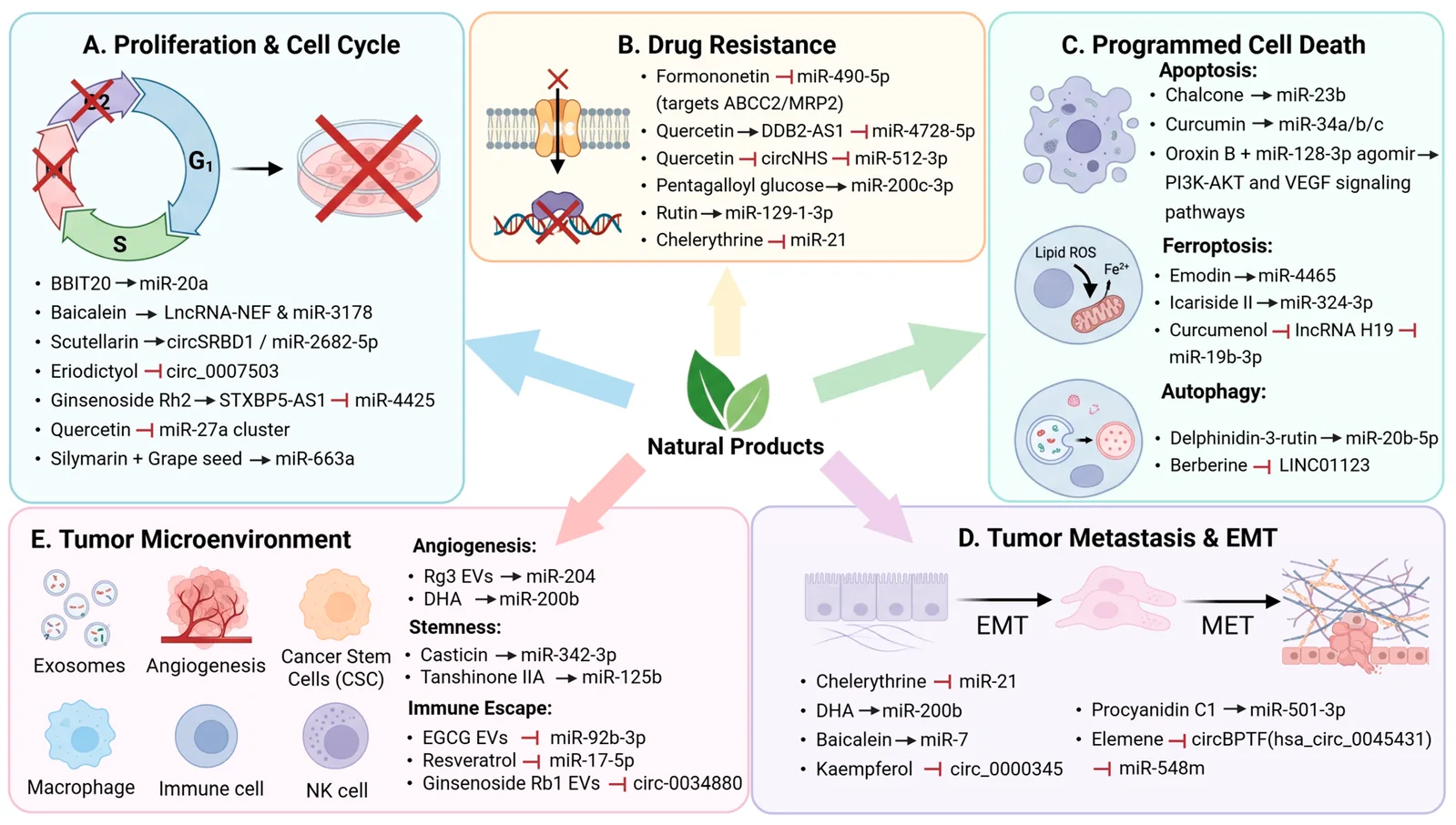
Multiple malignant phenotypes regulated by natural products via ncRNAs. Natural products remodel ncRNA networks to modulate five cancer hallmarks. (**A**) Proliferation & Cell Cycle: BBIT20, baicalein, scutellarin, and silymarin cause G1/S or M phase arrest via miR-20a, lncRNA-NEF/miR-3178, circSRBD1, and miR-663a. (**B**) Drug Resistance: Formononetin regulates ABCC2/MRP2 through miR-490-3p to improve chemosensitivity. (**C**) Programmed Cell Death: Chalcone, curcumin, oroxin B, and icariside II induce apoptosis, ferroptosis, and autophagy via ncRNA modulation. (**D**) Tumor Metastasis & EMT: Chelerythrine, dihydroartemisinin, and elemene suppress metastasis by regulating EMT-related ncRNAs. (**E**) Tumor Microenvironment: Related natural products inhibit angiogenesis, stemness, and immune escape through ncRNA regulation. These compound-ncRNA-target circuits enable phytochemicals to regulate malignant phenotypes. Created in BioRender. You, Q. (2026) https://BioRender.com/vu8g7k9.

Natural products have long served as a rich reservoir for anticancer drug discovery. A growing body of evidence reveals that their antitumor effects are frequently mediated through ncRNA regulation. This chapter reviews how natural product–ncRNA axes converge on key malignant phenotypes including proliferation, cell death, metastasis, microenvironment remodeling, and drug resistance (Figure 3, Table 2). Across the studies compiled in Table 2, polyphenols and flavonoids represent the most extensively investigated chemical classes, and the majority of the evidence derives from in vitro assays and xenograft models; only a minority of the studies include patient-derived or clinical data, and the therapeutic potential of these strategies therefore remains to be established in clinical settings. Representative natural products mentioned in this review, including their chemical classification, chemical structures, botanical sources, and PubChem compound identifiers, are summarized in Table 3.

### 4.1. Regulate ncRNA-Mediated Proliferation of Cancer Cells

Uncontrolled proliferative capacity is a key hallmark of cancer driven by aberrant cell cycle regulation and sustained replicative signalling. The cell cycle is influenced by a set of cyclins, cyclin-dependent kinases (CDKs), and checkpoint proteins to ensure orderly progression through the G1, S, G2, and M phases. In cancer, these regulatory mechanisms are often perturbed by mutations in tumour suppressors (p53, Rb), or overexpression of cyclins and CDKs, resulting in unscheduled proliferation and genomic instability. NcRNAs have emerged as central regulators of cell cycle progression, either as tumour suppressors (e.g., miR-34 family, miR-15/16) or oncogenes (e.g., miR-21). Natural products have been shown to restore cell cycle control through modulation of these ncRNA networks.

#### 4.1.1. Cell Cycle Arrest

Cell cycle arrest is an essential mechanism of natural products to inhibit the growth of cancer cells. These compounds can induce cell cycle checkpoint activation at G0/G1 or G2/M phases through up- or downregulation of specific ncRNAs. In pancreatic ductal adenocarcinoma (PDAC), the monoterpene indole alkaloid derivative BBIT20, a first-in-class suppressor of homologous recombination DNA repair, demonstrated potent antiproliferative activity regardless of BRCA mutation status. BBIT20 suppressed miR-20a, a miRNA associated with gemcitabine resistance, and induced G2/M cell cycle arrest and apoptosis. Such a dual mechanism of growth suppression and chemosensitization indicates the potential of DNA-repair-targeted natural compounds in PDAC therapy [127].

In osteosarcoma, baicalein derived from Scutellaria baicalensis was shown to suppress tumor cell growth by upregulating lncRNA-NEF (Neighboring Enhancer of FOXA2), which subsequently inactivated the Wnt/β-catenin pathway [18]. In HCC, baicalein similarly exhibited antiproliferative activity via miR-3178 upregulation, which directly targeted histone deacetylase 10 (HDAC10). This resulted in the inactivation of FoxO3α/p27/CDK2/Cyclin and E1AKT/MDM2/p53/Bcl2/Bax signalling pathways simultaneously, which effectively arrested cell cycle progression at the G1/S checkpoint [128]. In liver cancer cells, scutellarin induced G0/G1 phase arrest through the circSRBD1/miR-2682-5p/ESR1 pathway, which was validated by RNA immunoprecipitation and dual-luciferase reporter assays [129]. In breast carcinogenesis, eriodictyol, derived from citrus fruits, exerted potent inhibitory effects on cell transformation induced by environmental carcinogens in MCF10A cells. Mechanistic research showed that eriodictyol targets circ_0007503 to inhibit the PI3K/AKT axis. In vivo treatment of eriodictyol decreased the incidence of mammary tumours by 50% in carcinogen-treated rats, indicating its potential for chemoprevention [130].

Together, these findings suggest that natural products mediate the regulation of proliferative signalling via ncRNA expression to induce cell cycle arrest in various cancers. However, most studies are still preclinical, and further evaluation of pharmacokinetics, delivery, and safety is required before clinical application.

#### 4.1.2. Colony Formation and Viability

In addition to cell cycle arrest, the colony formation assay can be utilised to assess the long-term proliferative capability of cancer cells. The assay evaluates the capacity of single cells to form clonal colonies. Inhibition of colony formation is a more stringent measure of antiproliferative activity and is often mediated by ncRNA-regulated pathways affecting cell viability and clonogenic survival.

In breast cancer, ginsenoside Rh2 from ginseng was demonstrated to upregulate the lncRNA STXBP5-AS1 via promoter hypomethylation. STXBP5-AS1 sequesters oncogenic miR-4425 to derepress tumour suppressor RNF217. This mechanism led to a suppression of MCF-7 colony formation and an increase in apoptosis, suggesting an epigenetic-ncRNA interplay in Rh2 anticancer action [131]. In CRC, quercetin induced proteasomal degradation of the transcription factor Sp1, a master regulator of the miR-24-2/miR-23a/miR-27a cluster. MiR-27a was downregulated, which in turn led to reduced cell viability and promoted apoptosis [132]. In NSCLC, the combination of milk thistle silymarin extract and grape seed procyanidin extract suppressed cell proliferation through miR-663a upregulation synergistically, demonstrating the potential of combined natural product formulations [133].

Collectively, natural products inhibit colony formation and cell viability through several ncRNA-mediated mechanisms, including ceRNA networks, transcriptional repression of oncogenic miRNA clusters, and restoration of tumor-suppressive miRNA expression. The involvement of epigenetic modifications adds an additional level of regulatory complexity. These studies highlight the potential of natural compounds as adjuncts to conventional therapies, but the precise in vivo efficacy and tumour selectivity of these ncRNA-modulating approaches warrant further investigation.

### 4.2. Regulate ncRNA-Mediated Programmed Cell Death

Programmed cell death (PCD) includes regulated processes essential for tissue homeostasis, such as apoptosis, ferroptosis, pyroptosis, and autophagic cell death. Each PCD is characterised by specific molecular mechanisms, morphological features, and immunological consequences. Dysregulation of ncRNAs often enables cancer cells to escape from these death programs. Natural products can restore sensitivity to PCD through correction of ncRNA imbalance. Modulation of ncRNAs targeting specific pathways of PCD is a promising approach for overcoming apoptosis resistance in cancer.

#### 4.2.1. Apoptosis

Apoptosis, the most extensively studied type of PCD, occurs by two main pathways: the intrinsic pathway (mitochondrial), modulated by Bcl-2 family proteins that regulate Caspase 9 activation and Cytochrome c release, and the extrinsic pathway regulated by Fas/TRAIL receptors and Caspase 8. Both pathways converge to activate the effector caspase-3. ncRNAs, especially miRNAs, play key roles in regulating apoptotic gene expression at various levels, including Bcl-2 family members, death receptor expression, and caspase activity.

In breast and prostate cancer cells, the polyphenolic compound chalcone triggered ER stress-induced apoptosis through a distinctive NOX4-IRE1α sulfonation regulatory loop. Chalcone-activated NOX4 promoted IRE1α-dependent decay, selectively degrading miR-23b. The resulting miR-23b degradation further upregulated NOX4 expression, establishing a positive feedback loop that amplified the ER stress pathway and apoptosis [134]. Furthermore, Eriodictyol induced breast cancer apoptosis through circ_0007503 downregulation and PI3K/Akt inhibition [130]. In CRC, curcumin enhanced the ROS/KEAP1/ARE/NRF2 signaling cascade, leading to NRF2-dependent transactivation of miR-34b/c and miR-34a via direct binding to antioxidant response elements in their promoter regions. This induced apoptosis and cellular senescence in a p53-independent manner, with genetic deletion of miR-34a/b/c significantly abrogating curcumin’s pro-apoptotic efficacy [16]. In HCC, a chitosan-based nanoparticle co-delivering Oroxin B and miR-128-3p agomir synergistically induced apoptosis by simultaneously interfering with PI3K-AKT and VEGF signaling pathways, achieving superior antitumor efficacy compared to either monotherapy and demonstrating the potential of nanoparticle-based combined delivery of natural products and ncRNA therapeutics [135]. Moreover, Baicalein induced HCC cell apoptosis through the miR-3178/HDAC10/AKT axis [128]. Scutellarin activated apoptotic cascades in liver cancer through the circSRBD1/miR-2682-5p/ESR1 network [129].

Collectively, these studies illustrate that natural products engage the apoptotic machinery through diverse ncRNA-mediated mechanisms, including relieving the repression of pro-apoptotic miRNAs, disrupting oncogenic ceRNA networks, restoring p53-independent apoptotic pathways, and activating ER stress-mediated cell death.

#### 4.2.2. Ferroptosis

Ferroptosis is a non-apoptotic form of PCD driven by iron-dependent lipid peroxidation and characterized by ROS accumulation and GSH depletion [156]. Unlike apoptosis, ferroptosis does not require caspase activation and is morphologically distinguished by mitochondrial shrinkage and increased membrane density [157,158]. Glutathione peroxidase 4 (GPX4) acts as the central negative regulator of ferroptosis by converting lipid peroxides into non-toxic lipid alcohols, and its activity is tightly regulated by the cysteine/glutathione antiporter system xCT [159,160].

Recently, ncRNAs have emerged as significant regulators of ferroptosis sensitivity by modulating GPX4 and iron metabolism genes. In HCC, emodin induced ferroptosis through upregulation of miR-4465, which directly targeted NFE2L3 and subsequently suppressed the HMGCR/GPX4 axis, resulting in massive accumulation of ROS, iron overload, and GPX4 depletion [136]. In renal cell carcinoma (RCC), icariside II upregulated miR-324-3p, which directly targeted the 3′UTR of GPX4 mRNA, resulting in GPX4 downregulation. The administration of ICS II led to Fe2+ accumulation, increased malondialdehyde and ROS levels, and GSH reduction, thereby contributing to the induction of ferroptosis in RCC cells, with a concomitant suppression of their proliferative, migratory, and invasive capabilities [137]. In NSCLC, curcumenol from Curcuma wenyujin was identified as a potent ferroptosis inducer. Through RNA sequencing analysis, curcumenol was found to significantly downregulate lncRNA H19, which normally sequesters miR-19b-3p to suppress ferritin heavy chain 1 (FTH1), a key negative regulator of ferroptosis that sequesters intracellular iron. This caused an increased labile iron pool, increased lipid peroxidation, and finally ferroptosis in both in vitro and xenograft models. LncRNA H19 overexpression effectively rescued the ferroptosis phenotype, confirming the essential role of the ncRNA pathway [138].

The emerging role of ferroptosis in cancer therapy, particularly through ncRNA-mediated regulation of GPX4 and iron metabolism, represents a promising avenue for the treatment of apoptosis-resistant tumors. However, the potential off-target effects of systemic ferroptosis induction on normal tissues, the mechanisms by which natural products selectively trigger ferroptosis in cancer cells, and the long-term safety of ferroptosis-based therapies require careful investigation in future studies.

#### 4.2.3. Pyroptosis

Pyroptosis is a pro-inflammatory type of PCD modulated by the gasdermin protein family, particularly gasdermin D (GSDMD). Upon cleavage by inflammatory caspases (caspase-1/4/5/11), the N-terminal domain of GSDMD oligomerizes to generate plasma membrane pores, resulting in cell swelling, membrane rupture, and the release of pro-inflammatory cytokines such as IL-1β and IL-18 [161,162]. Pyroptosis has garnered increasing attention as a mechanism that bridges cell death with antitumor immunity, as its immunogenic nature can potentiate antitumor immune responses [163].

In HCC, a recent structural optimization study of the natural product (-)-Gomisin M1 (GM) led to the synthesis of 37 diphenyl amine ester derivatives, among which derivative 13j demonstrated enhanced binding affinity to the TRBP-Dicer complex, a key regulator of miRNA biogenesis. By disrupting TRBP-Dicer interaction, 13j globally modulated miRNA biosynthesis and simultaneously induced both apoptosis and pyroptosis in HCC cells while hindering the epithelial–mesenchymal transition (EMT) process. With favorable drug-like properties and low systemic toxicity, 13j provides a promising scaffold for the development of miRNA-based HCC therapeutics [139].

The crosstalk between pyroptosis and ncRNA biology remains largely unexplored. Given the immunogenic nature of pyroptosis and its potential to promote antitumor immunity through the release of DAMPs, future studies should investigate the combination of natural products that induce pyroptosis with immune checkpoint inhibitors and other immunotherapeutic approaches for synergistic anticancer efficacy.

#### 4.2.4. Cuproptosis and Parthanatos

Cuproptosis is a recently identified form of PCD triggered by copper overload, in which copper directly binds to lipoylated components of the tricarboxylic acid (TCA) cycle, such as DLAT and LIAS, causing protein aggregation, loss of iron–sulfur cluster proteins, and proteotoxic stress in an FDX1-dependent manner [164,165]. Emerging evidence indicates that ncRNAs modulate cuproptosis sensitivity; for instance, cuproptosis-related lncRNA signatures have been established as prognostic models in non-small cell lung cancer [166]. Although direct evidence of natural products regulating cuproptosis through ncRNAs remains limited, natural compounds such as triptolide have been shown to induce cuproptosis in cervical cancer cells [167], suggesting that the ncRNA–cuproptosis axis may represent an unexplored target of natural products.

Parthanatos is another regulated cell death pathway initiated by hyperactivation of poly(ADP-ribose) polymerase 1 (PARP1), which generates poly(ADP-ribose) (PAR) polymers that promote mitochondrial release and nuclear translocation of apoptosis-inducing factor (AIF), culminating in large-scale DNA fragmentation [168]. The nuclear protein NUPR1 has been identified as a key suppressor of parthanatos by counteracting hyperPARylation-dependent cell death [169], and natural product-derived NUPR1 inhibitors have recently shown anticancer activity [170]; however, ncRNA-mediated regulation of parthanatos in the context of natural products remains largely unexplored. Future studies should investigate whether natural products can sensitize cancer cells to cuproptosis and parthanatos through ncRNA networks, thereby broadening the PCD-targeting repertoire of phytochemicals beyond apoptosis, ferroptosis, pyroptosis, and autophagy.

#### 4.2.5. Autophagy

Autophagy is a catabolic process that delivers cytoplasmic constituents to lysosomes for degradation. As a double-edged sword in malignancy, it can increase survival under stress conditions, but excessive or prolonged autophagy results in a non-apoptotic form of cell death known as autophagic cell death or type II PCD [171]. The expression of crucial autophagy-related genes (ATGs), many of which are regulated by ncRNAs, affects the balance between cytoprotective and cytotoxic autophagy [172].

In glioma, delphinidin-3-rutin from purple sweet potato induced cytostatic autophagy by upregulating miR-20b-5p to suppress Atg7, an essential autophagy-related E1-like enzyme [140]. In ovarian cancer, berberine simultaneously inhibited two interconnected metabolic processes, autophagy and glycolysis, which are critical for cancer cell survival, via inhibition of the LINC01123/P65 interaction. Berberine reduced P65 protein stability and impaired MAPK10 transcription by disrupting the physical association between LINC01123 and P65 [20].

These findings emphasise the complexity of the regulation of autophagy by natural products and ncRNAs. Autophagy in cancer can be either a survival mechanism or a cell death pathway, depending on the context, making the approach of targeting autophagy therapeutically complicated. Future studies should focus on identifying the molecular determinants that distinguish cytoprotective from cytotoxic autophagy, and on developing strategies to selectively engage the latter in cancer therapy.

### 4.3. Regulate ncRNA-Mediated Tumor Metastasis and Invasion

The vast majority of cancer-related deaths are triggered by metastasis, which is the spread of cancer cells from the original tumour to distant organs. Local invasion, intravasation, circulation survival, extravasation, and colonisation at distant locations make up the metastatic cascade [173,174]. NcRNAs act as master switches in the metastatic cascade, regulating each step and coordinating the expression of EMT transcription factors and metastatic effector genes.

EMT, a crucial first stage that provides cancer cells the ability to migrate and invade, is marked by the overexpression of mesenchymal markers (N-cadherin, vimentin, ZEB1, Snail) and the downregulation of epithelial markers (E-cadherin). In nasopharyngeal carcinoma, chelerythrine reversed EMT and inhibited migration by transcriptionally suppressing pri-miR-21, restoring PTEN and inactivating PI3K/AKT signaling [141]. In CRC, curcumin suppressed lung metastasis formation through induction of the ROS/NRF2/KEAP1/miR-34a/b/c pathway, leading to mesenchymal-to-epithelial transition (MET), a process opposite to EMT. In vivo imaging confirmed that curcumin treatment significantly reduced lung metastases in NOD/SCID mice in a miR-34a-dependent manner [16]. In ovarian cancer, dihydroartemisinin (DHA) inhibited peritoneal dissemination by upregulating miR-200b, a master regulator of the epithelial phenotype that concurrently suppressed BMI-1 and VEGF-A. The clinical significance of this axis was confirmed by clinical research, which showed that dual positivity for BMI-1 and VEGF-A in patient tumour samples was linked to poor progression-free survival [142].

In osteosarcoma, baicalein inhibited invasion and metastasis through lncRNA-NEF upregulation and Wnt/β-catenin inactivation [18], while in GC, the same compound suppressed migration through the miR-7/FAK/AKT pathway [143]. In CRC, kaempferol inhibited metastasis by downregulating circ_0000345, attenuating JMJD2C/β-catenin signaling [144]. In colon cancer, procyanidin C1 suppressed tumor growth and metastasis by upregulating miR-501-3p to target HIGD1A [145]. In HCC, elemene suppressed lung metastasis by downregulating circBPTF (hsa_circ_0045431), a pro-metastatic circRNA that functions as a molecular sponge for miR-548m, thereby activating POLR3G and ZBTB41. Elemene inhibited circBPTF biogenesis by suppressing the RNA-binding proteins HNRNPU and HNRNPC, disrupting this oncogenic axis [146].

### 4.4. Regulate ncRNA-Mediated Remodeling of Tumor Microenvironment

Cancer cells, stromal cells (cancer-associated fibroblasts, endothelial cells), immune cells (T lymphocytes, macrophages, NK cells, dendritic cells), and extracellular matrix components constitute the TME, a complex ecosystem that is essential to the development of tumours and the response to treatment. NcRNAs secreted by tumor cells via exosomes or regulated within TME components significantly influence angiogenesis, cancer stem cell maintenance, metabolic reprogramming, and immune surveillance. Natural products can reshape the TME through the modulation of these ncRNA networks, offering opportunities to overcome the immunosuppressive and therapy-resistant features of the TME.

#### 4.4.1. Tumor Angiogenesis

Angiogenesis, the generation of novel blood vessels from pre-existing vasculature, is crucial for tumor progression beyond a certain size and is primarily driven by VEGF signaling. Vasculogenic mimicry (VM), an alternative mechanism in which tumor cells themselves generate vessel-like structures in a manner independent of endothelial cells, contributes to resistance to conventional anti-angiogenic therapy and is associated with unfavorable prognosis.

In ovarian cancer, DHA inhibited VEGF-A/VEGFR2-mediated neovascularization through miR-200b upregulation, thereby reducing tumor vasculature [142]. In pancreatic ductal adenocarcinoma (PDAC), ginsenoside Rg3 employed a novel exosome-mediated strategy to suppress both angiogenesis and VM. Rg3 increased miR-204 levels in PDAC cell-derived exosomes, which were taken up by recipient cells to downregulate DVL3, impairing stemness-dependent VM formation. Rg3 also inhibited HUVEC tube formation, demonstrating a dual anti-angiogenic effect that addresses the limitations of conventional anti-VEGF monotherapy [19].

#### 4.4.2. Cancer Stem Cell Stemness

Cancer stem cells (CSCs) are a subpopulation of cancer cells with self-renewal ability and multilineage differentiation potential, and are considered to contribute to cancer initiation, recurrence, and therapeutic resistance [175,176]. Key stemness-associated transcription factors such as Nanog, Sox2, and Oct4, along with surface markers including CD133 and CD44 [177], are regulated by intricate ncRNA networks that integrate extrinsic and intrinsic signals. In cervical cancer, Casticin (CAS) upregulated miR-342-3p, leading to the downregulation of stemness markers, including CD133, CD49f, Nanog, and Sox2 [147]. In breast cancer, tanshinone IIA reduced cancer stemness via miR-125b downregulation and upregulation, thereby reducing the CD24^−^/CD44^+^ subpopulation and enhancing adriamycin sensitivity, demonstrating a functional association between stemness and chemoresistance [148].

Multiple natural products targeting the regulation of stemness-associated ncRNA axes highlight the potential of targeting CSCs via ncRNA modulation. However, the plasticity of CSC phenotypes and redundancy of stemness signalling pathways present major challenges. Combinatorial strategies targeting multiple ncRNA-stemness axes simultaneously may be required for effective eradication of CSCs and prevention of tumour recurrence.

#### 4.4.3. Tumor Metabolism

One well-known characteristic of cancer is metabolic reprogramming, particularly the transition to aerobic glycolysis (Warburg effect). It provides the necessary biosynthetic precursors and ATP to support the rapid proliferation of tumour cells [178]. Increased expression of glucose transporters and glycolytic enzymes, which are frequently regulated by carcinogenic transcription factors including HIF-1α and c-Myc, mediates this metabolic reprogramming [179,180]. NcRNAs have become significant modulators of the expression of metabolic enzymes and of signalling pathways that control metabolic flux in cancer cells.

In ovarian cancer, berberine suppressed glycolysis via the LINC01123/P65/MAPK10 pathway. By disrupting the LINC01123-P65 interaction, berberine reduced P65 protein stability and impaired its transcriptional activation of MAPK10, thereby decreasing glycolytic flux. This metabolic suppression, combined with reduced autophagic flux, contributed to the inhibition of ovarian cancer cell proliferation and metastasis [20]. Given the limited studies on ncRNA-mediated metabolic regulation by natural products, future work should expand the repertoire of metabolic targets, including fatty acid oxidation, glutamine metabolism, and nucleotide biosynthesis, to provide a more comprehensive picture of the metabolic consequences of ncRNA modulation.

#### 4.4.4. Tumor Immune Escape

Tumours leverage various mechanisms to escape immune surveillance, such as recruitment of immunosuppressive cells (Myeloid-derived suppressor cells, regulatory T cells, M2-polarized tumour-associated macrophages), deactivation of the antigen presentation mechanism, and production of immunosuppressive cytokines, e.g., IL-10 and TGF-β [181,182]. ncRNAs are important players in modulating the immunogenicity of the TME by affecting the expression of immune checkpoints, molecules involved in the antigen presentation process, and cytokine signalling molecules.

In GC, EGCG from green tea inhibited M2 macrophage polarisation through STAT3 targeting to transcriptionally repress PLXNC1, blocking oncogenic miR-92b-5p transfer via tumor-derived exosomes [17]. In breast cancer, resveratrol increased the NK cell-modulated cytotoxicity by downregulating miR-17-5p, a direct target of MINK1, leading to the activation of the MINK1/JNK/c-Jun axis and upregulation of NKG2D ligand ULBP2 on breast cancer cells [149]. In CRC liver metastasis, ginsenoside Rb1 was shown to inhibit pre-metastatic niche (PMN) formation by reducing the biogenesis of circ-0034880 in tumor-derived EVs. Circ-0034880-enriched TEVs normally promote the activation of SPP1-high CD206+ pro-tumor macrophages in the liver, creating an immunosuppressive microenvironment for metastatic colonisation. Ginsenoside Rb1 inhibited PMN formation and subsequent liver metastasis by suppressing circ-0034880 [150].

The studies show that natural products can remodel the TME via diverse ncRNA mechanisms to modulate macrophage polarisation, improve NK cell activity, stimulate DC maturation, and prevent PMN formation. The development of nanoparticle-based co-delivery systems is a particularly promising approach for combined ncRNA-natural product immunotherapy. Future studies should aim to understand how these immunomodulatory effects can be optimally combined with immune checkpoint inhibitors to achieve durable antitumor responses.

### 4.5. Regulate ncRNA-Mediated Tumor Drug Resistance

Drug resistance (intrinsic or acquired) continues to be one of the major challenges in clinical oncology. Resistance mechanisms include increased drug efflux by ATP-binding cassette (ABC) transporters, increased DNA repair capacity, evasion of apoptosis, activation of alternative survival signalling pathways, and metabolic adaptation [183,184]. NcRNAs have emerged as important modulators of drug resistance by targeting drug transporters, DNA repair enzymes, regulators of apoptosis, and stemness-related factors. Natural products can reverse these ncRNA imbalances and restore drug sensitivity.

In nasopharyngeal carcinoma, chelerythrine targeted the miR-21/PTEN/PI3K/AKT axis, re-expressing PTEN to counteract PI3K/AKT hyperactivation driving therapeutic resistance [141]. In CRC, formononetin exhibited synergy with 5-fluorouracil by up-regulating miR-490-3p, which targeted the 3′UTR of ABCC2 encoding the multidrug resistance protein MRP2. The importance of this ncRNA axis was further confirmed by the improved sensitivity to formononetin in MRP2 knockout or miR-490-3p overexpression [151]. In TNBC, pentagalloyl glucose reversed doxorubicin resistance through multiple complementary mechanisms, including restoration of miR-200c-3p expression. It reversed EMT through downregulation of ZEB1, vimentin, P-gp, and MRP-1 and upregulation of E-cadherin [152].

Quercetin demonstrated dual chemosensitizing effects across different cancer types. In NSCLC, it reversed cisplatin resistance via the DDB2-AS1/miR-4728-5p/p53 pathway. The precise molecular mechanism involved DDB2-AS1 sponging miR-4728-5p to activate p53 and inhibit the xCT/GPX4 ferroptosis defence system [153]. In prostate cancer, quercetin increased radiosensitivity by targeting the radiation-induced ARv7-mediated circNHS/miR-512-5p/XRCC5 pathway that efficiently re-sensitized cancer cells to radiation therapy [154]. In breast cancer, rutin increased the sensitivity of pirarubicin (THP) and alleviated THP-induced cardiotoxicity at the same time. Rutin upregulated miR-129-1-3p, directly targeting GRIN2D, and attenuated oxidative stress and calcium overload in cardiac and tumour tissues, as an example of theranostic adjuvant therapy [155].

The field of ncRNA-dependent reversal of drug resistance by natural products is rapidly expanding with promising translational potential. Nevertheless, the heterogeneity of resistance mechanisms between patients and within tumours underscores the need for biomarker-based strategies to identify the best candidates for specific natural product-ncRNA interventions. Further work is additionally required to determine whether early combination with natural products can delay the development of resistance to drugs and not simply reverse it after resistance has been established. It should be emphasized that all of the resistance-reversal effects described above represent preclinical chemosensitization observed in cell-based or xenograft models; no natural product–ncRNA combination has yet demonstrated reversal of drug resistance in clinical trials. Moreover, for several compounds (e.g., curcumin), the concentrations required to achieve these effects in vitro exceed the plasma concentrations attainable after oral administration, underscoring the need for pharmacokinetic optimization and careful dose–exposure assessment before clinical extrapolation.

## 5. Non-Coding RNAs as Biomarkers and Targets for Natural Product Anticancer Therapy

**Figure 4:**
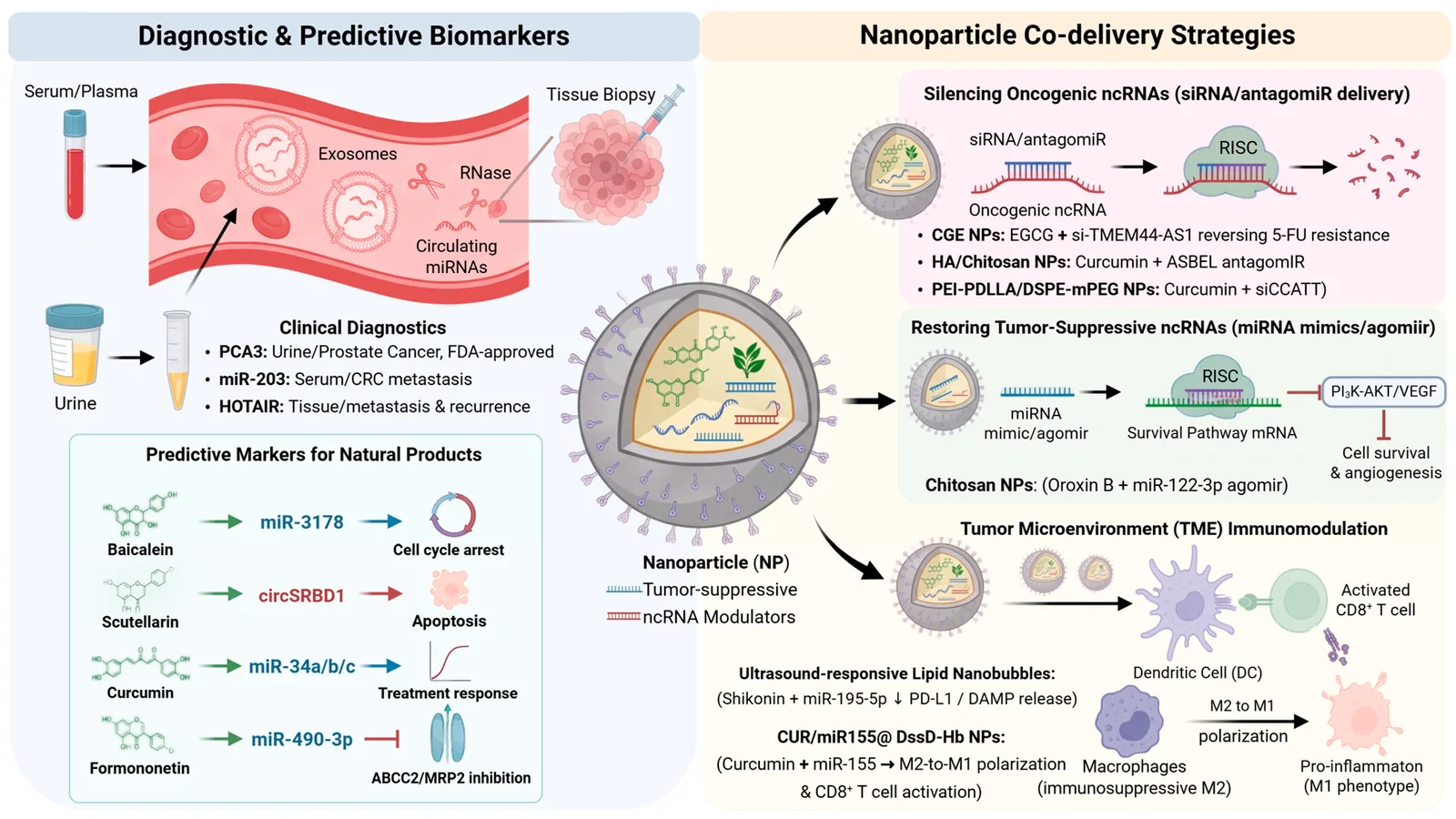
NcRNAs as biomarkers and co-delivery targets in natural-product anticancer therapy. NcRNAs serve as minimally invasive biomarkers in exosomes, urine, and tissues, such as PCA3, miR-203, and HOTAIR; specific ncRNAs can predict natural product efficacy. Three nanoparticle co-delivery strategies are applied: nanoparticles carrying siRNA/antagomiR silence oncogenic ncRNAs to reverse chemotherapy resistance; carriers delivering miRNA mimics restore tumor-suppressive ncRNAs and related signaling; TME-targeted immunomodulatory nanoparticles remodel macrophages and activate CD8^+^ T cells, combining ncRNA theranostics with natural product anti-tumor therapy. Created in BioRender. You, Q. (2026) https://BioRender.com/bt7ghj6.

Beyond their mechanistic contributions to tumor progression, ncRNAs hold considerable promise as diagnostic biomarkers and therapeutic targets in natural product-based anticancer strategies. This section discusses the clinical utility of ncRNAs as minimally invasive biomarkers and highlights nanoparticle co-delivery platforms that integrate natural products with ncRNA-directed therapeutics (Figure 4).

### 5.1. Diagnostic and Prognostic Value of ncRNAs

NcRNAs exhibit tissue-specific and cancer-specific expression patterns, endowing them with the potential as biomarkers for cancer diagnosis and prognosis. PCA3, a prostate-specific lncRNA detectable in urine, represents the first FDA-approved ncRNA biomarker for cancer diagnosis. Circulating miRNAs, protected by exosomes or protein complexes from RNase degradation, have demonstrated particular clinical utility [185,186]. For instance, elevated serum miR-203 levels in CRC patients correlate with increased risk of liver and systemic metastases [187]. Similarly, HOTAIR overexpression in tumor tissues has been linked to metastasis, recurrence, and unfavorable prognosis across breast, colon, and liver cancers [188].

Within the scope of natural product research, ncRNAs serve as valuable surrogate markers for therapeutic response and disease stratification. In HCC, miR-3178 upregulation by baicalein and circSRBD1 downregulation by scutellarin both correlate with improved cell cycle arrest and apoptosis, suggesting their potential as pharmacodynamic biomarkers [128,129]. In CRC, curcumin-induced miR-34a/b/c upregulation serves as both a mechanistic indicator and a predictor of treatment response [16], while formononetin efficacy is closely linked to miR-490-3p levels, which inversely correlate with ABCC2/MRP2 expression in clinical CRC specimens [151]. These examples illustrate that ncRNAs not only function as diagnostic and prognostic biomarkers but also as predictive indicators for natural product-based therapies, bridging mechanistic understanding with clinical application. Most candidate biomarkers discussed here are experimental candidates identified in preclinical models or single-center studies; none has been clinically validated for the indications described, and prospective multi-center validation with standardized assays is required before clinical application.

### 5.2. Targeted Therapy Strategies Targeting ncRNAs

Currently available delivery platforms for RNA therapeutics can be broadly divided into viral and non-viral systems, including lipid nanoparticles (LNPs), GalNAc-conjugated siRNAs, polymeric and inorganic nanoparticles, extracellular vesicle (EV)-based carriers, and viral vectors [189,190]. Viral platforms, including adeno-associated virus (AAV) vectors and oncolytic viruses, enable durable transgene expression but raise concerns regarding immunogenicity and potential insertional mutagenesis [21]. Beyond RNA delivery, the delivery of natural products themselves represents a distinct challenge: most polyphenols and flavonoids (e.g., curcumin, quercetin, baicalein) have poor aqueous solubility, rapid metabolic conjugation, and low oral bioavailability, and several terpenoids and alkaloids face similar pharmacokinetic limitations. Established formulation strategies—including prodrug design, nanocrystals, polymeric micelles, cyclodextrin inclusion complexes, self-emulsifying drug delivery systems, phospholipid complexes (phytosomes), and metal coordination—have been applied to improve the solubility and systemic exposure of phytochemicals [6,7]. In this context, the co-delivery platforms described below offer a dual solution: nanocarriers simultaneously solubilize poorly water-soluble natural products and protect labile ncRNA therapeutics from nuclease degradation, addressing the twin bioavailability barriers of both payload classes.

Suicide gene therapy represents an additional strategy in which a delivered enzyme gene converts a non-toxic prodrug into a cytotoxic agent within tumor cells, as exemplified by herpes simplex virus thymidine kinase (HSV-TK) with ganciclovir (GCV), or cytosine deaminase with 5-fluorocytosine (5-FC) [191]. Its therapeutic efficacy is amplified by the bystander killing effect, whereby cytotoxic metabolites or death signals are transferred from transduced to neighboring untransduced tumor cells through connexin 43 (Cx43)-containing gap junctions, apoptotic bodies, and extracellular vesicles. Notably, ncRNAs are increasingly integrated into this paradigm: microRNA-regulated oncolytic viruses and miRNA-detargeted vectors restrict transgene expression to tumor cells, thereby improving tumor specificity and safety [192,193]. Conversely, ncRNAs can modulate bystander killing by regulating gap junction proteins and EV-mediated intercellular communication. Future studies should investigate whether natural products can enhance bystander killing through ncRNA-dependent regulation of gap junctional communication, an underexplored but promising avenue for combination therapy in cancer.

Rapid degradation, inadequate cellular uptake, and off-target effects restrict the clinical translation of ncRNA therapeutics. Nanoparticle delivery systems can overcome these barriers, and co-delivery with natural products complementing ncRNA modulation provides synergistic advantages. Current strategies include silencing oncogenic ncRNAs or restoring tumor-suppressive ncRNA functions.

One major approach is delivery of siRNAs or antagomiRs to silence oncogenic ncRNAs. For GC, Zhou et al. developed chitosan-gelatin-EGCG nanoparticles (CGE NPs) that deliver siRNA targeting lncRNA TMEM44-AS1, which sponges miR-2355-5p to upregulate PPP1R13L and inhibit P53 signaling. The CGE nanocarrier containing green tea polyphenol EGCG demonstrated enhanced gene silencing efficiency and synergistically reversed 5-FU resistance in GC xenografts [194]. For TNBC, He et al. designed hyaluronic acid/chitosan-based nanocomplexes that co-delivered the lncRNA ASBEL antagomiR and curcumin, enabling near-infrared fluorescence imaging and modulating the ASBEL/BTG3 and Bcl-2/c-Met pathways to inhibit tumour growth and metastasis [195]. For CRC, Jia et al. constructed PEI-PDLLA/DSPE-mPEG hybrid NPs co-delivering siCCAT1 and curcumin, which showed excellent tumour inhibition in vivo [196].

Another significant approach is the delivery of tumour-suppressive miRNA mimics in combination with natural products, complementary to silencing. For HCC, Wang et al. developed chitosan-based nanoparticles carrying the miR-128-3p agomir and the flavonoid Oroxin B, which simultaneously inhibit the PI3K-AKT and VEGF pathways [135]. Furthermore, Tan et al. developed ultrasound-responsive lipid nanobubbles co-encapsulating miR-195-5p and shikonin for HCC immunotherapy. miR-195-5p targeted PD-L1 directly, mimicking immune checkpoint blockade, while shikonin induced immunogenic cell death and DAMP release. This coordination reprogrammed the TME to promote infiltration of CD8^+^ T cells [197].

The immunomodulatory potential of ncRNA delivery can be further amplified through co-delivery with natural products targeting complementary immune pathways. A ROS/glutathione dual-sensitive nanoparticle co-delivering curcumin and miR-155 (CUR/miR155@DssD-Hb NPs) stimulated dendritic cell maturation, promoted CD8^+^ T cell activation, drove M2-to-M1 macrophage polarization, and simultaneously reduced immunosuppressive populations (MDSCs, Tregs, M2 TAMs). This comprehensive immune reprogramming resulted in durable antitumor immunity and reduced lung metastasis in melanoma and TNBC models [198].

These co-delivery strategies can not only avoid the limitations of ncRNA therapeutics and natural products alone, but also synergise anti-tumor effects. The variety of platforms, from chitosan-based nanoparticles to polymeric hybrid systems and stimulus-responsive nanobubbles, illustrates the increasing sophistication in the design of nanocarriers. Future optimisation of targeting specificity, release kinetics, and scalability will be critical for clinical translation. Despite their promise, the clinical translation of these nanoplatforms faces unresolved challenges, including carrier-related toxicity and immunogenicity, insufficient in vivo stability, batch-to-batch reproducibility, scalable manufacturing under GMP conditions, and regulatory approval pathways, all of which require systematic evaluation.

## 6. Limitations of Non-Coding RNAs in Cancer Therapeutics

Despite the promising preclinical evidence summarized above, several limitations currently constrain the clinical translation of ncRNA-based cancer therapeutics. First, naked ncRNAs are intrinsically unstable, being rapidly degraded by nucleases and cleared from circulation, while natural products often suffer from poor bioavailability–dual barriers that nanoparticle-based co-delivery platforms are only beginning to overcome [21,189]. Second, off-target silencing and dose-limiting toxicities, including innate immune stimulation and nanoparticle-associated hepatotoxicity, remain major safety concerns [21]. Third, the function of a given ncRNA is highly context-dependent: the same ncRNA can exert opposing oncogenic or tumor-suppressive roles depending on tumor type, disease stage, or microenvironmental context, as reported for circHIPK3, miR-149-5p, and miR-221-5p, which cautions against one-size-fits-all strategies. Fourth, mechanistic evidence is often incomplete; although most functional studies rely on the ceRNA hypothesis, quantitative modeling suggests that many circRNAs are expressed at copy numbers too low to effectively sequester miRNAs, underscoring the need for rigorous validation of ncRNA–miRNA–mRNA interactions [199]. Finally, clinical-grade evidence remains scarce, and regulatory, manufacturing, and delivery hurdles must be resolved before ncRNA therapeutics can enter routine oncology practice.

## 7. Future Perspectives

The regulatory functions of natural products on various ncRNA classes in various cancer types, including the modulation of miRNAs, lncRNAs, circRNAs, tsRNAs, piRNAs, and snoRNAs, have been compiled in this review. These ncRNA-mediated mechanisms support the inhibition of malignant phenotypes, such as uncontrolled proliferation, evasion of PCD, metastatic dissemination, TME remodelling, and therapeutic resistance. The translational potential of these findings is further highlighted by the application of ncRNAs as diagnostic and prognostic biomarkers and the emergence of nanoparticle-based platforms for co-delivery of natural products and ncRNA therapeutics. Despite these advances, several critical challenges need to be addressed before natural product-ncRNA-based strategies can be translated into clinical practice.

A major bottleneck is the mechanistic understanding of how natural products modulate ncRNA expression. Although most functional studies are based on the ceRNA hypothesis, quantitative modelling suggests that most circRNAs are expressed at copy numbers too low to effectively sequester miRNAs. In addition to sponging, ncRNA–protein interactions and direct ncRNA-epigenetic machinery crosstalk are emerging regulatory layers that deserve systematic investigation. Recent advances in CLIP-seq, cryo-electron microscopy, and AI-based prediction models have significantly expedited the discovery of ncRNA–protein interactions, while single-cell ncRNA profiling and spatial transcriptomics now allow context-specific mapping of ncRNA expression across TMEs. An integration of these approaches will be critical to move beyond descriptive correlative studies toward a mechanistic understanding of how natural products reprogram ncRNA networks.

The twin problems of inadequate bioavailability of natural products and intrinsic instability of ncRNAs continue to represent a major translational bottleneck. Fortunately, recent advances in co-delivery nanoplatforms have demonstrated the feasibility of overcoming both limitations simultaneously. For example, ROS/glutathione dual-sensitive nanoparticles co-loaded with curcumin and miR-155 induced comprehensive immune reprogramming by eliciting dendritic cell maturation, stimulating CD8^+^ T cell activation, and driving M2-to-M1 macrophage polarisation while simultaneously inhibiting immunosuppressive populations. Simultaneously, aptamer-RIBOTAC strategies have emerged as a precision tool for tumor-specific ncRNA degradation. Engineered circular RNA technologies are making great strides beyond conventional delivery vehicles. The optimised IRES-containing circRNA constructs resulted in higher translation efficiency than linear mRNA. In vitro circularisation approaches using ribozyme-based systems now allow for the scalable synthesis of synthetic circRNAs. In addition, the LNP and exosome-based platforms are actively being explored for tumor-targeted delivery of ncRNA. In the future, efforts should be directed towards systematic optimisation of nanoparticle targeting specificity, controlled release kinetics and scalability for clinical-grade manufacturing.

The duality of ncRNA functions is also context-dependent, adding another layer of complexity. The same ncRNA may exert opposing functions depending on the type of tumour, disease stage, or microenvironmental context, as reported for circHIPK3, miR-149-5p, miR-221-5p, and many others. This dichotomy cautions against one-size-fits-all approaches and highlights the value of targeted, conditionally active interventions. Emerging solutions include the design of TME-responsive ncRNA therapeutics. For example, circRNA vectors can be designed with activation elements gated by hypoxia, pH, or reactive oxygen species. At the same time, the development of single-cell and spatial transcriptomics is making it possible to build cell-type-resolved ncRNA atlases for different types of cancer, providing a basis for patient-specific target selection. These high-resolution datasets can be used to train machine learning models that predict individual ncRNA vulnerabilities, enabling a shift from broad-spectrum to precision-guided natural product–ncRNA combination therapies.

Several emerging modalities are expanding the therapeutic repertoire of ncRNA-based cancer therapy beyond the conventional silencing or replacement paradigms. An alternative approach is to use small activating RNAs (saRNAs). miRNA therapeutics silence aberrantly expressed genes, whereas saRNAs selectively upregulate transcription of dormant tumour suppressor genes. This approach has entered a phase I clinical trial for liver cancer and requires further study of its combination with natural products that modulate upstream regulatory pathways. Another frontier is circular RNA vaccines. A circRNA vaccine against GPC3 recently demonstrated potent adaptive anti-tumor immunity in HCC models. The intersection of ncRNA engineering and gene editing is also gaining traction. Embedding prime editing guide RNAs in circRNA scaffolds could allow scarless genome correction in malignant cells. These developments, along with the chemical versatility of natural products, suggest a future where natural products will be used not only as ncRNA modulators, but also as a core component of rationally designed multi-modal therapeutic regimens.

In particular, the cross-talk of natural product–ncRNA regulation with cancer immunotherapy is a promising frontier. Recent studies have shown that miR-34a conjugated to anti-CD47 antibodies can deliver targeted ncRNA and inhibit immune checkpoints in triple-negative breast cancer. Nanocomposite hydrogels that capture and use tumor-derived miRNAs for photothermal immunotherapy demonstrate the versatility of ncRNA-based immune modulation [124]. Natural products can also induce immunogenic cell death (ICD), and miRNA-mediated DAMP regulation offers a promising but unexplored way to boost anti-tumor immunity. Future studies should systematically map the intersections of natural product-ncRNA axes with key immune checkpoints, ICD pathways, and tumor-associated macrophage polarisation, thereby establishing a rational framework for designing combination regimens integrating natural products, ncRNA modulation, and immunotherapy.

Future translational efforts should also address the standardization of natural products and extracts—including chemical fingerprinting, marker compound quantification, and batch-to-batch consistency—as well as rigorous pharmacokinetic characterization of pharmacologically achievable doses and exposures, systematic safety and toxicity assessment, and stepwise validation in animal models before clinical trials. Reproducibility of natural product–ncRNA mechanisms across independent laboratories should be established prior to clinical development.

In summary, although many hurdles remain for clinical translation, the integration of advanced delivery systems, multi-omics profiling, and increasing knowledge of ncRNA functions provide a promising outlook for the development of natural product-derived cancer therapeutics.

## 8. Conclusions

In conclusion, natural products exert broad anticancer effects through the regulation of multiple ncRNA classes, modulating key malignant phenotypes including proliferation, programmed cell death, metastasis, tumor microenvironment remodeling, and therapeutic resistance. The convergence of ncRNA biology with natural product pharmacology has opened new opportunities for biomarker development and for co-delivery strategies in which nanoparticle platforms simultaneously transport phytochemicals and ncRNA therapeutics. Nevertheless, the overwhelming majority of the evidence summarized in this review derives from preclinical cell-based and animal studies, and no natural product–ncRNA strategy has yet been validated in clinical settings. Clinical translation will require rigorous pharmacokinetic and pharmacodynamic characterization, standardized natural product preparations, clinically validated biomarkers, and well-designed clinical trials. Future research should therefore prioritize mechanistic depth, evidence quality, and clinical feasibility over descriptive breadth.

## Figures and Tables

**Table 1:** Comparison of the major ncRNA classes.

RNA Class	Origin	Structure	Biogenesis	Target	Mechanism	Duration of Effect
miRNA	Endogenous genome; synthetic mimics available	Single-stranded, 19–25 nt	Pol II → pri-miRNA → Drosha/DGCR8 → pre-miRNA → Exportin-5 → Dicer → RISC loading	mRNAs (mainly 3′UTR; also 5′UTR/CDS/promoter)	Translational repression; mRNA decay; nuclear transcriptional regulation	Relatively stable (hours–days)
lncRNA	Intergenic, antisense, intronic, enhancer regions	Linear, >200 nt, spliced/polyadenylated	Pol II transcription with co-transcriptional processing	Chromatin, proteins, mRNAs, miRNAs	Chromatin remodeling; transcriptional regulation; ceRNA/miRNA sponging; scaffolding; micropeptide encoding	Highly variable: short-lived to very stable
circRNA	Back-splicing of pre-mRNA (exonic, intronic, mitochondrial)	Covalently closed loop	Back-splicing (intron-pairing, RBP-mediated, lariat-driven)	miRNAs, RBPs, mRNAs, DNA, ribosomes	miRNA sponging; protein scaffold/decoy; transcriptional regulation; circR-loops; IRES/m6A-driven translation	Very stable (exonuclease-resistant)
tsRNA/tRF	Mature tRNAs or tRNA precursors	Fragments of 14–40 nt (tRF-1/3/5; tiRNA 30–40 nt)	Angiogenin cleavage (tiRNA) or Dicer/nuclease cleavage (tRF)	mRNAs, translation machinery, retrotransposons	miRNA-like silencing; translation inhibition; stress responses	Modest; dynamically regulated by stress
piRNA	piRNA clusters (transposon-rich loci)	24–31 nt, 2′-O-methylated 3′ end	Primary processing plus ping-pong amplification; loaded onto PIWI proteins	Transposon transcripts, mRNAs, chromatin	Transposon silencing (DNA/histone methylation, slicer cleavage); gene regulation	Stable (2′-O-methylation)
snoRNA	Introns of host genes (e.g., SNHG loci)	60–300 nt; H/ACA or C/D box	Processed from introns; nucleolar localization with partner proteins	rRNAs and other RNAs; splicing factors	rRNA pseudouridylation/2′-O-methylation; splicing regulation; sdRNA generation	Stable; nucleolar localization

**Table 2:** Natural products modulate ncRNAs to regulate malignant phenotypes in multiple cancers.

NcRNA	Cancer Type	Expression	Natural Product	Chemical Class	Natural Source	Evidence	Function	Biological Roles	Ref.
Proliferation: Cell cycle arrest									
miR-20a	PDAC	↓	BBIT20	—	—	In vitro	Suppress miR-20a	Cell cycle arrest ↑, Apoptosis ↑	[127]
lncRNA-NEF	OS	↑	Baicalein	Flavonoid	Scutellaria baicalensis	In vitro; in vivo	Inactivate Wnt/β-catenin signaling	Tumor growth ↓, Invasion ↓, Metastasis ↓	[18]
miR-3178	HCC	↑	Baicalein	Flavonoid	Scutellaria baicalensis	In vitro	Target HDAC10, Inactivate AKT/MDM2/p53/Bcl2/Bax, FoxO3a/p27/CDK2/Cyclin E1	Proliferation ↓, Colony formation ↓, Cell cycle arrest ↑, Apoptosis ↑	[128]
circSRBD1	HCC	↓	Scutellarin	Flavonoid	Scutellaria baicalensis	In vitro	Sponge miR-2682-5p, Upregulate ESR1	Proliferation ↓, Cell cycle arrest ↑, Apoptosis ↑	[129]
circ_0007503	BC	↓	Eriodictyol	Flavonoid	Citrus fruits	In vitro; in vivo	Repress the PI3K/Akt pathway	Viability ↓, Apoptosis ↑, Carcinogenesis ↓	[130]
Proliferation: Colony formation and viability									
STXBP5-AS1	BC	↑	Ginsenoside Rh2	Triterpenoid saponin	Panax ginseng	In vitro	Sponge miR-4425, Upregulate RNF217	Colony formation ↓, Apoptosis ↑	[131]
miR-27a	CRC	↓	Quercetin	Flavonoid	Onion, tea, apple	In vitro	Degrade Sp1 via proteasome, Disrupt Sp1-miR-27a axis	Proliferation ↓, Apoptosis ↑	[132]
miR-663a	NSCLC	↑	Silymarin + grape seed procyanidin	Flavonolignan; proanthocyanidin	Silybum marianum; Vitis vinifera	In vitro	Upregulate miR-663a	Proliferation ↓	[133]
Programmed cell death: Apoptosis									
miR-23b	BC, PCa	↓	Chalcone	Polyphenol	Various plants	In vitro; in vivo	Activate NOX4-IRE1α sulfonation-RIDD axis, Degrade miR-23b	Apoptosis ↑ (ER stress), Tumor growth ↓	[134]
circ_0007503	BC	↓	Eriodictyol	Flavonoid	Citrus fruits	In vitro	Repress the PI3K/Akt pathway	Apoptosis ↑	[130]
miR-34a, miR-34b/c	CRC	↑	Curcumin	Polyphenol	Curcuma longa	In vitro; in vivo	Activate ROS/KEAP1/NRF2/ARE, NRF2 binds ARE motifs to induce miR-34a/b/c	Apoptosis ↑, Senescence ↑	[16]
miR-128-3p	HCC	↑	Oroxin B	Flavonoid	Oroxylum indicum	In vitro	Chitosan-nanoparticle co-delivery, Interfere PI3K-AKT and VEGF signaling	Apoptosis ↑	[135]
miR-3178	HCC	↑	Baicalein	Flavonoid	Scutellaria baicalensis	In vitro	Target HDAC10, Inactivate AKT/MDM2/p53/Bcl2/Bax, FoxO3a/p27/CDK2/Cyclin E1	Apoptosis ↑	[128]
circSRBD1	HCC	↓	Scutellarin	Flavonoid	Scutellaria baicalensis	In vitro	Sponge miR-2682-5p, Upregulate ESR1	Apoptosis ↑	[129]
Programmed cell death: Ferroptosis									
miR-4465	HCC	↑	Emodin	Anthraquinone	Rheum palmatum; Polygonum cuspidatum	In vitro	Target NFE2L3, Suppress HMGCR/GPX4 axis	ROS ↑, Iron overload ↑, Ferroptosis ↑	[136]
miR-324-3p	RCC	↑	Icariside II	Flavonoid	Epimedium species	In vitro	Target GPX4 3′UTR	Ferroptosis ↑, Proliferation ↓, Migration ↓, Invasion ↓	[137]
lncRNA H19, miR-19b-3p	NSCLC	H19 ↓, miR-19b-3p ↑	Curcumenol	Sesquiterpenoid	Curcuma species	In vitro	H19 ceRNA sponges miR-19b-3p, Suppress FTH1	Ferroptosis ↑, Proliferation ↓	[138]
Programmed cell death: Pyroptosis									
miRNA biogenesis	HCC	Modulation	Gomisin M1 derivative 13j	Lignan	Schisandra chinensis	In vitro	Target TRBP-Dicer complex	Apoptosis ↑, Pyroptosis ↑	[139]
Programmed cell death: Autophagy									
miR-20b-5p	GLI	↑	Delphinidin-3-rutin	Anthocyanin	Elderberry and other berries	In vitro	Target Atg7, Induce Atg7-dependent cytostatic autophagy	Proliferation ↓, Autophagic cell death ↑	[140]
LINC01123	OC	↓	Berberine	Alkaloid	Coptis chinensis; Berberis	In vitro; in vivo	Inhibit LINC01123/P65 interaction, Suppress MAPK10	Autophagy ↓, Glycolysis ↓, Proliferation ↓, Metastasis ↓	[20]
Metastasis and invasion									
miR-21	NPC	↓	Chelerythrine	Alkaloid	Chelidonium majus; Zanthoxylum	In vitro	Transcriptionally suppress pri-miR-21, Restore PTEN, Inactivate PI3K/AKT	EMT reversal ↑, Migration ↓, Invasion ↓	[141]
miR-34a, miR-34b/c	CRC	↑	Curcumin	Polyphenol	Curcuma longa	In vitro; in vivo	Activate ROS/KEAP1/NRF2/miR-34a/b/c cascade, Induce MET	Lung metastasis ↓	[16]
miR-200b	OC	↑	DHA	Sesquiterpene lactone	Artemisia annua	In vitro; in vivo	Target BMI-1 and VEGF-A	Peritoneal dissemination ↓, Metastasis ↓	[142]
lncRNA-NEF	OS	↑	Baicalein	Flavonoid	Scutellaria baicalensis	In vitro; in vivo	Inactivate Wnt/β-catenin signaling	Invasion ↓, Metastasis ↓	[18]
miR-7	GC	↑	Baicalein	Flavonoid	Scutellaria baicalensis	In vitro	Target FAK/AKT	Migration ↓	[143]
circ_0000345	CRC	↓	Kaempferol	Flavonoid	Tea, broccoli, fruits	In vitro; in vivo	Attenuate JMJD2C/β-catenin signaling	Metastasis ↓, Invasion ↓	[144]
miR-501-3p	COC	↑	Procyanidin C1	Proanthocyanidin	Grape seed; cinnamon	In vitro; in vivo	Target HIGD1A, Suppress HIGD1A expression	Tumor growth ↓, Metastasis ↓	[145]
circBPTF	HCC	↓	Elemene	Sesquiterpenoid	Curcuma species	In vitro; in vivo	Sponge miR-548m, Suppress POLR3G/ZBTB41	Lung metastasis ↓	[146]
Tumor microenvironment: Angiogenesis									
Exosomal miR-204	PDAC	↑	Ginsenoside Rg3	Triterpenoid saponin	Panax ginseng	In vitro; in vivo	Target DVL3, Impair stemness, Inhibit VM	VM ↓, Angiogenesis ↓, Stemness ↓, Tumor growth ↓	[19]
miR-21	NPC	↓	Chelerythrine	Alkaloid	Chelidonium majus; Zanthoxylum	In vitro	Transcriptionally suppress pri-miR-21, Restore PTEN, Inactivate PI3K/AKT	EMT reversal ↑, Migration ↓, Invasion ↓	[141]
Tumor microenvironment: Cancer stem cell stemness									
miR-342-3p	CC	↑	Casticin	Flavonoid	Vitex species	In vitro; in vivo	Target FoxM1, Downregulate CD133/CD49f/Nanog/Sox2	Stemness ↓, Sphere ↓, Colony ↓, Tumorigenicity ↓	[147]
miR-125b	BC	↓	Tanshinone IIA	Diterpenoid	Salvia miltiorrhiza	In vitro	Upregulate STARD13, Inactivate miR-125b/STARD13	Stemness ↓, Sphere ↓, CD24^−^/CD44^+^↓, Adriamycin sensitivity ↑	[148]
Tumor microenvironment: Metabolism									
LINC01123	OC	↓	Berberine	Alkaloid	Coptis chinensis; Berberis	In vitro; in vivo	Inhibit LINC01123/P65 interaction, Suppress MAPK10	Glycolysis ↓, Autophagy ↓, Proliferation ↓, Metastasis ↓	[20]
Tumor microenvironment: Immune escape									
Exosomal miR-92b-5p	GC	↓	EGCG	Polyphenol (catechin)	Camellia sinensis	In vitro; in vivo	Target STAT3, Repress PLXNC1, Inhibit M2 polarization	M2 polarization ↓, Immune escape ↓	[17]
miR-17-5p	BC	↓	Resveratrol	Polyphenol (stilbene)	Vitis vinifera; Polygonum cuspidatum	In vitro; in vivo	Activate MINK1/JNK/c-Jun, Upregulate ULBP2	NK cytotoxicity ↑, Immune eradication ↑	[149]
circ-0034880	CRC	↓	Ginsenoside Rb1	Triterpenoid saponin	Panax ginseng	In vitro; in vivo	Suppress SPP1hiCD206^+^ pro-tumor macrophages	PMN formation ↓, Liver metastasis ↓	[150]
Drug resistance									
miR-21	NPC	↓	Chelerythrine	Alkaloid	Chelidonium majus; Zanthoxylum	In vitro	Restore PTEN, Inactivate PI3K/AKT	Therapeutic resistance ↓	[141]
miR-490-3p	CRC	↑	Formononetin	Isoflavonoid	Astragalus membranaceus; Trifolium pratense	In vitro	Target ABCC2 3′UTR, Downregulate MRP2, Synergize with 5-FU	5-FU sensitivity ↑	[151]
miR-200c-3p	TNBC	↑	Pentagalloyl glucose	Polyphenol (gallotannin)	Paeonia; Rhus species	In vitro	Reverse EMT, Suppress P-gp/MRP-1, Increase DOX accumulation	DOX resistance ↓, Apoptosis ↑, EMT reversal ↑	[152]
DDB2-AS1, miR-4728-5p	NSCLC	↑	Quercetin	Flavonoid	Onion, tea, apple	In vitro	Sponge miR-4728-5p, Activate p53, Inhibit xCT/GPX4	Cisplatin resistance ↓, Ferroptosis ↑	[153]
circNHS, miR-512-5p	PCa	↑	Quercetin	Flavonoid	Onion, tea, apple	In vitro; in vivo	Target radiation-induced ARv7-mediated circNHS/miR-512-5p/XRCC5 signaling	Radiosensitivity ↑, Tumor progression ↓	[154]
miR-129-1-3p	BC	↑	Rutin	Flavonoid (flavonol glycoside)	Sophora japonica; buckwheat	In vitro; in vivo	Target GRIN2D, Attenuate oxidative stress/calcium overload	THP sensitivity ↑, Cardiotoxicity ↓, Metastasis ↓	[155]

Abbreviations: PDAC, pancreatic ductal adenocarcinoma; OS, osteosarcoma; HCC, hepatocellular carcinoma; BC, breast cancer; CRC, colorectal cancer; NSCLC, non-small cell lung cancer; RCC, renal cell carcinoma; GLI, glioma; OC, ovarian cancer; NPC, nasopharyngeal carcinoma; GC, gastric cancer; COC, colon cancer; CC, cervical cancer; TNBC, triple-negative breast cancer; PCa, prostate cancer; VM, vasculogenic mimicry; EMT, epithelial–mesenchymal transition; NK, natural killer; PMN, pre-metastatic niche; 5-FU, 5-fluorouracil; DOX, doxorubicin; THP, pirarubicin. Explanation: Arrow up (↑) represents increased expression, activated signaling or enhanced biological function; arrow down (↓) represents decreased expression, inhibited signaling or attenuated biological function.

**Table 3:** Representative natural products (chemical classification, chemical structure, and natural source).

Natural Product	Chemical Class	Chemical Structure	Natural Source	PubChem CID
Baicalein	Flavonoid (flavone)	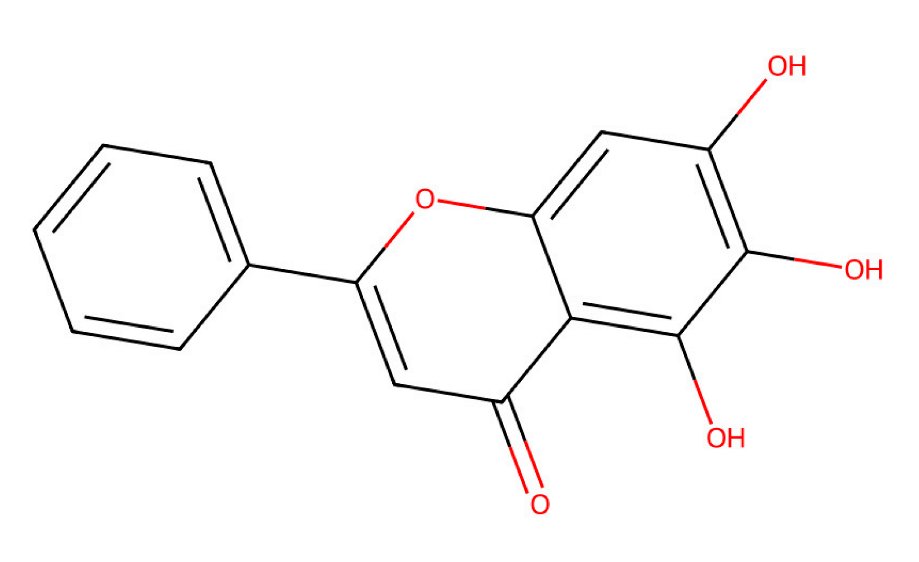	Scutellaria baicalensis	5281605
Scutellarin	Flavonoid (flavone glucuronide)	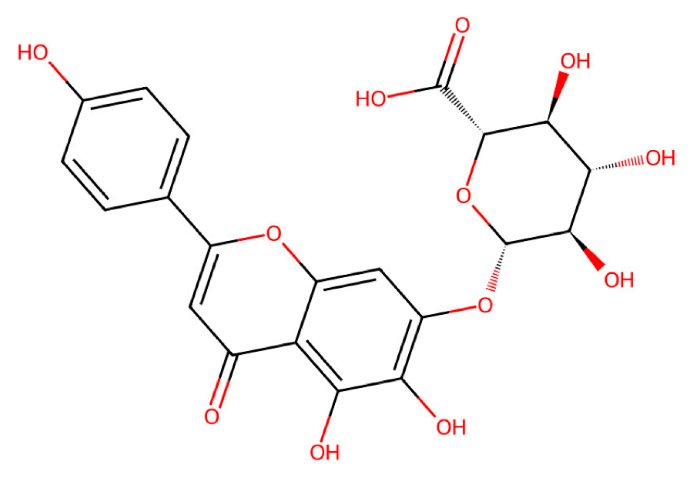	Scutellaria baicalensis	185617
Eriodictyol	Flavonoid (flavanone)	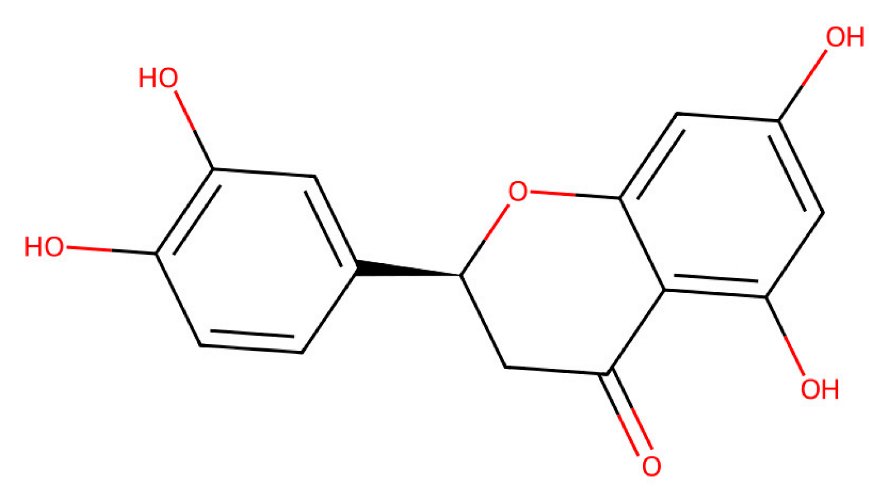	Citrus fruits	440735
Quercetin	Flavonoid (flavonol)	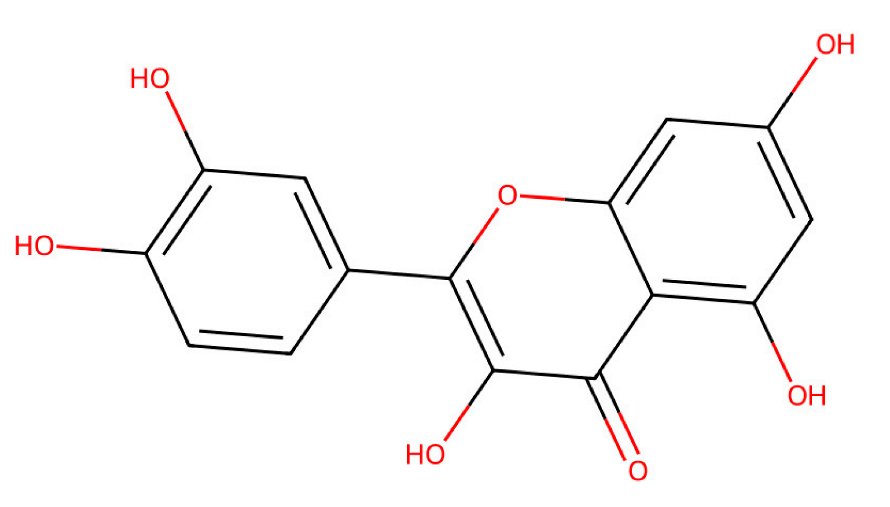	Onion, tea, apple	5280343
Kaempferol	Flavonoid (flavonol)	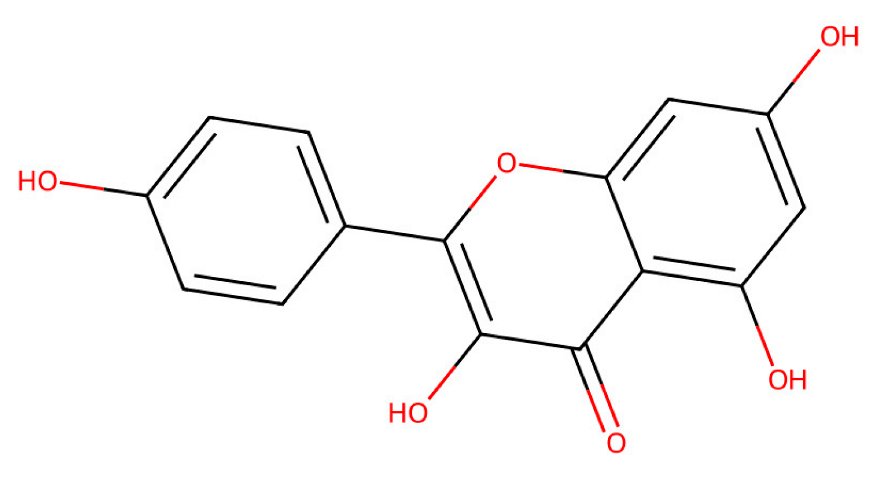	Tea, broccoli, fruits	5280863
Rutin	Flavonoid (flavonol glycoside)	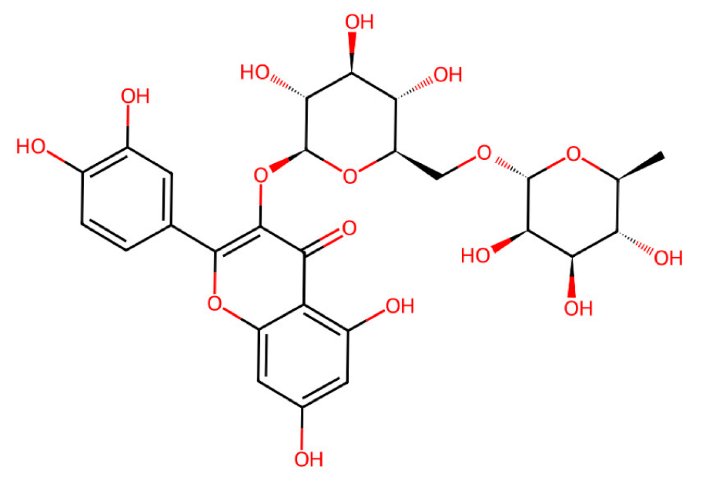	Sophora japonica; buckwheat	5280805
Casticin	Flavonoid (flavonol methyl ether)	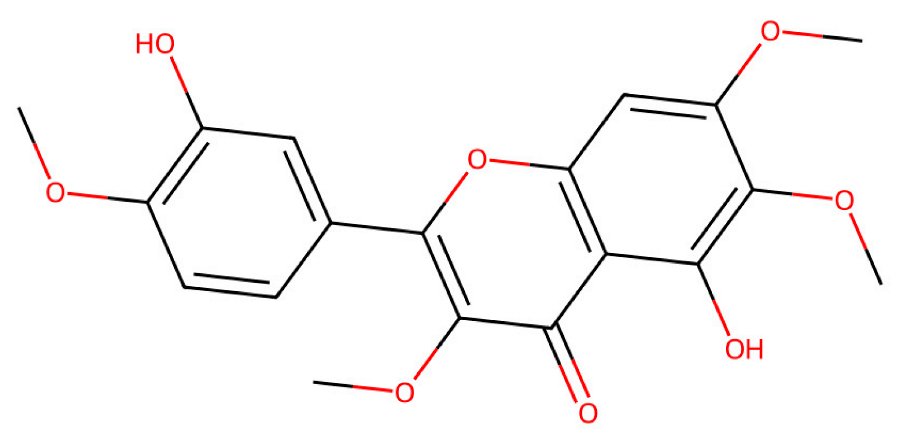	Vitex species	5315263
Icariside II	Flavonoid (prenylated flavonol glycoside)	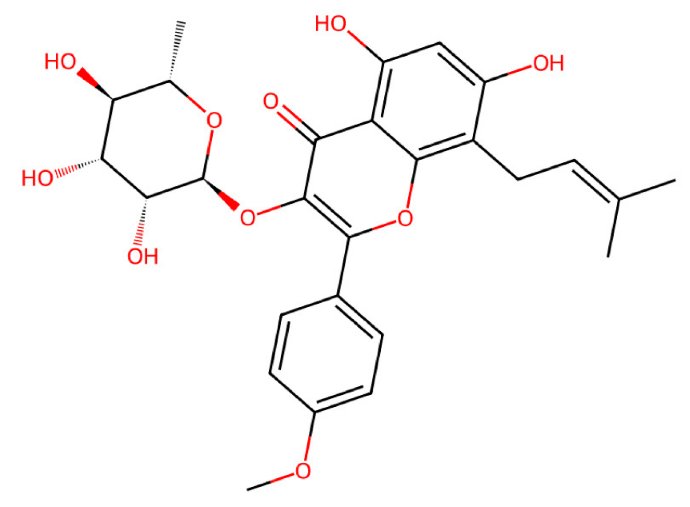	Epimedium species	5488822
Formononetin	Isoflavonoid	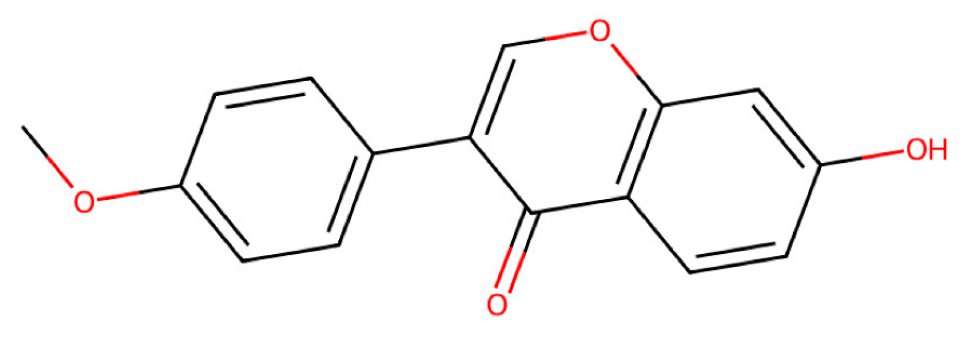	Astragalus membranaceus; Trifolium pratense	5280378
Oroxin B	Flavonoid (flavone diglucoside)	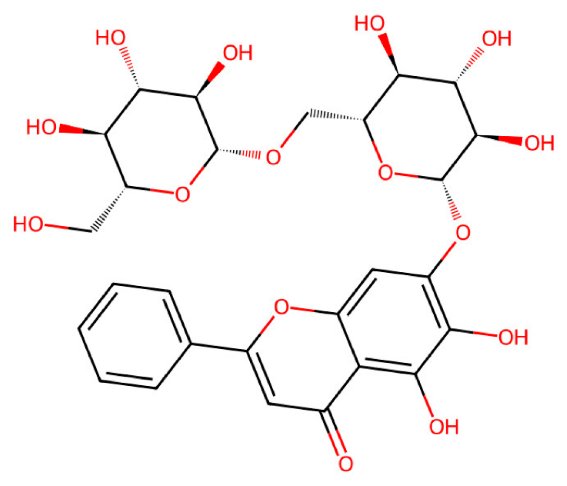	Oroxylum indicum	10077207
Delphinidin-3-rutin	Anthocyanin	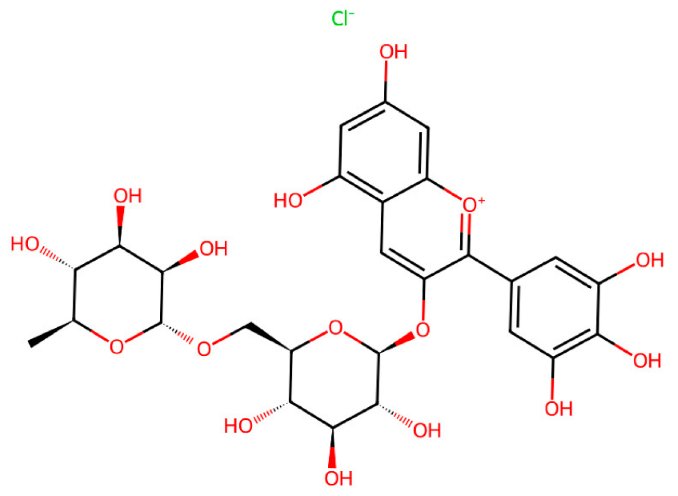	Elderberry and other berries	192918
Curcumin	Polyphenol (curcuminoid)	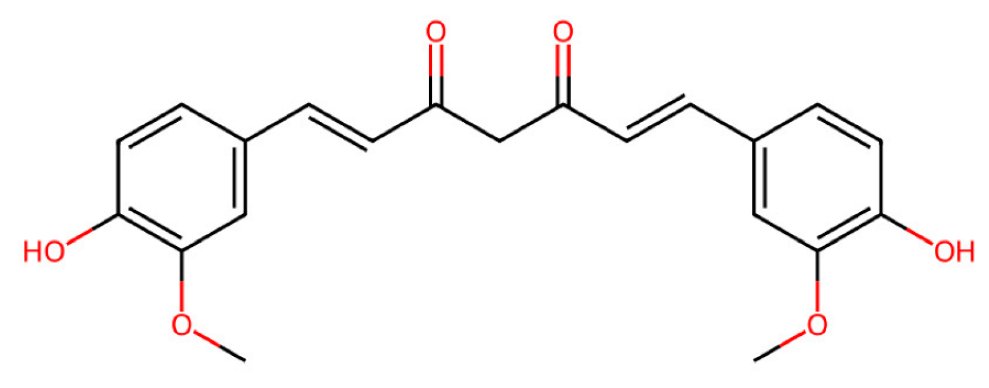	Curcuma longa	969516
Resveratrol	Polyphenol (stilbene)	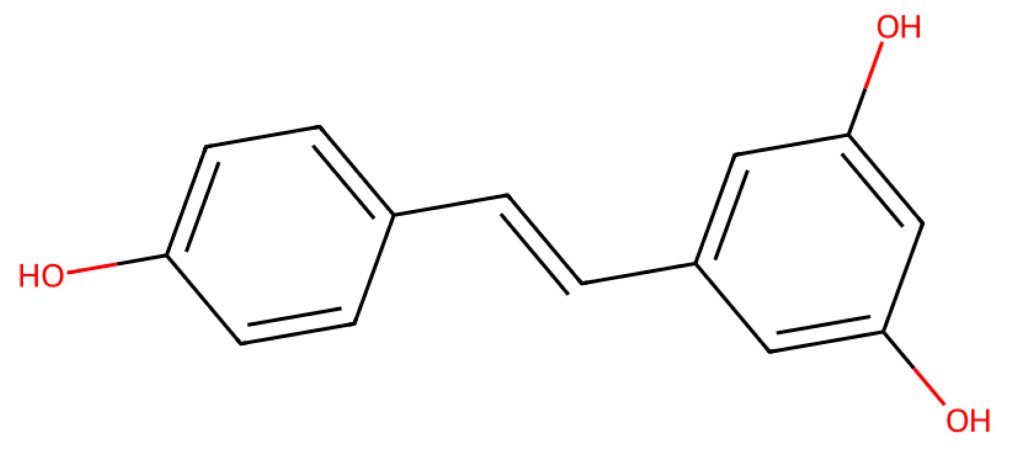	Vitis vinifera; Polygonum cuspidatum	445154
EGCG	Polyphenol (flavan-3-ol/catechin)	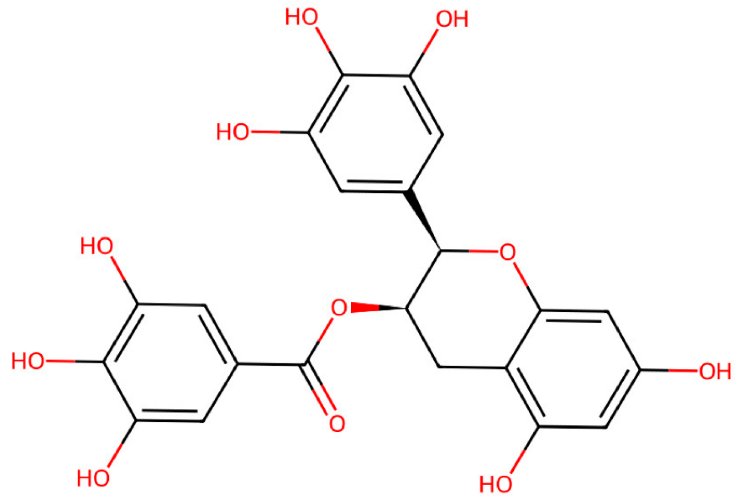	Camellia sinensis	65064
Chalcone	Polyphenol (chalcone)	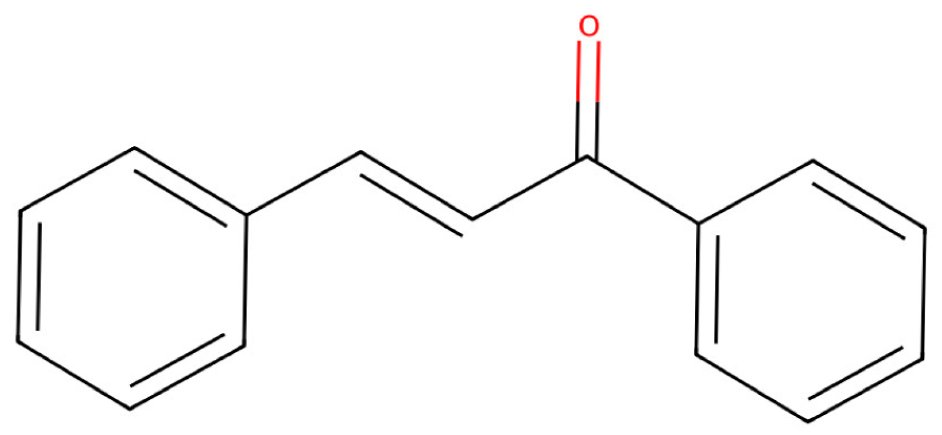	Various plants	637760
Pentagalloyl glucose	Polyphenol (gallotannin)	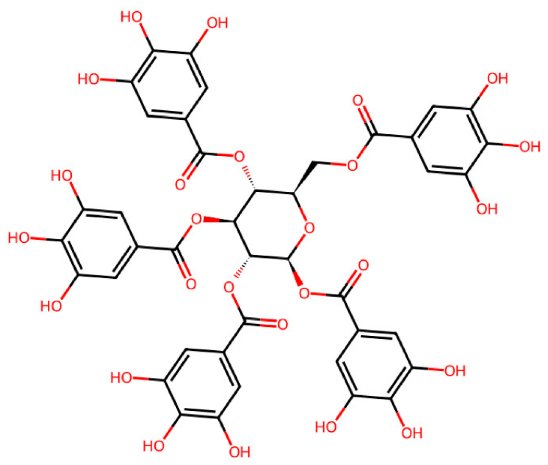	Paeonia; Rhus species	65238
Silibinin (from silymarin)	Flavonolignan	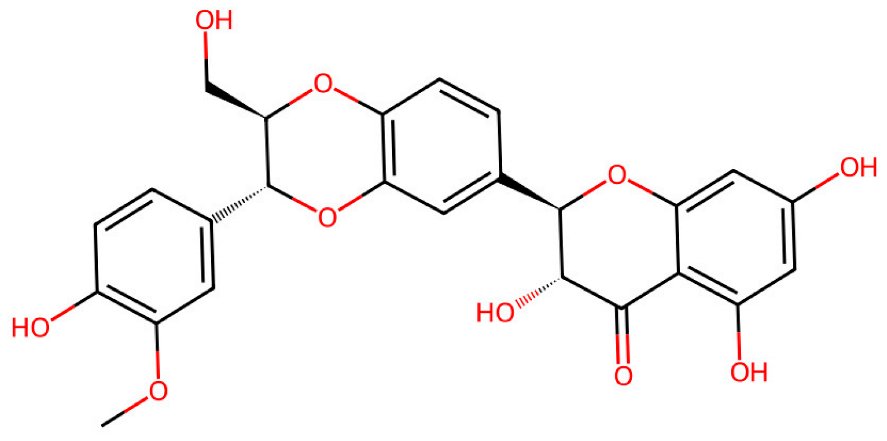	Silybum marianum	31553
Procyanidin B2	Proanthocyanidin	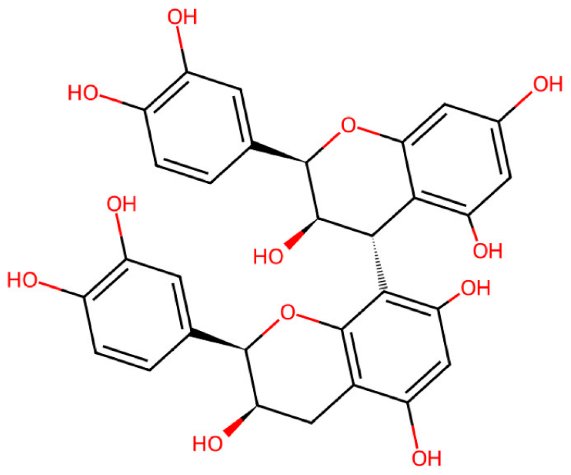	Grape seed; Vitis vinifera	122738
Procyanidin C1	Proanthocyanidin	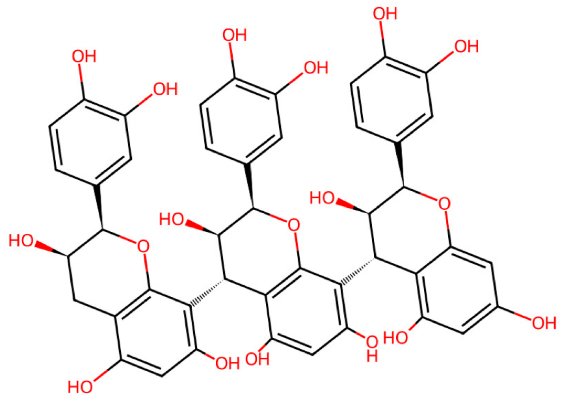	Grape seed; cinnamon	169853
Emodin	Anthraquinone	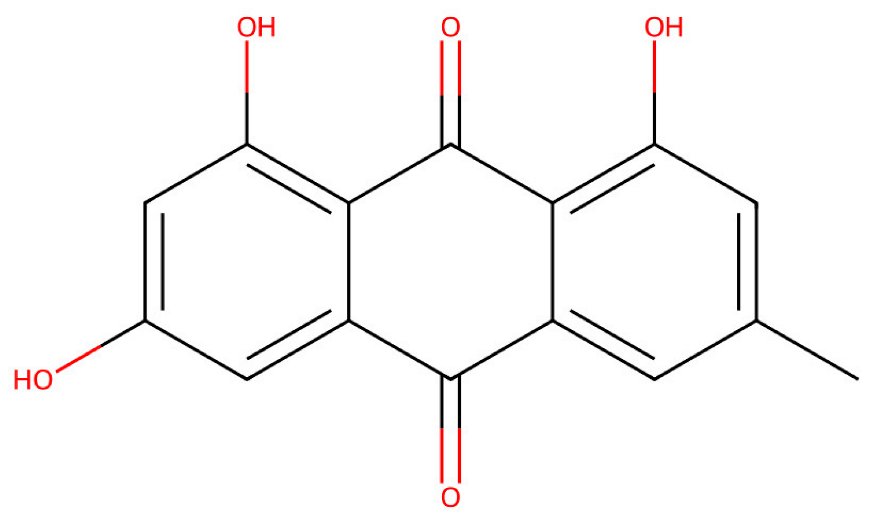	Rheum palmatum; Polygonum cuspidatum	3220
Ginsenoside Rg3	Triterpenoid saponin	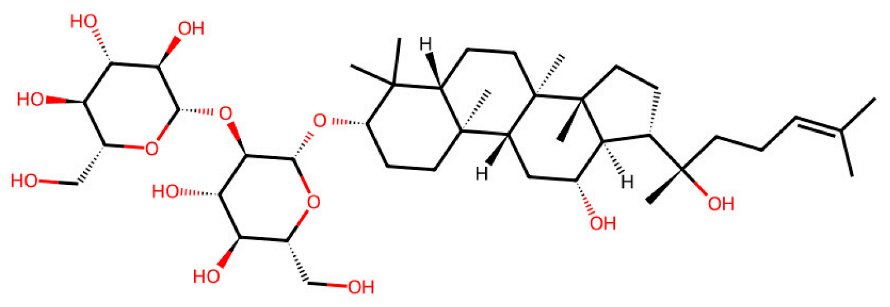	Panax ginseng	9918693
Ginsenoside Rh2	Triterpenoid saponin	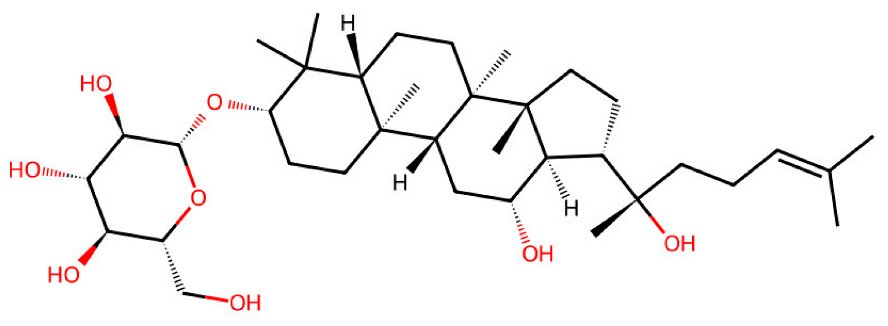	Panax ginseng	119307
Ginsenoside Rb1	Triterpenoid saponin	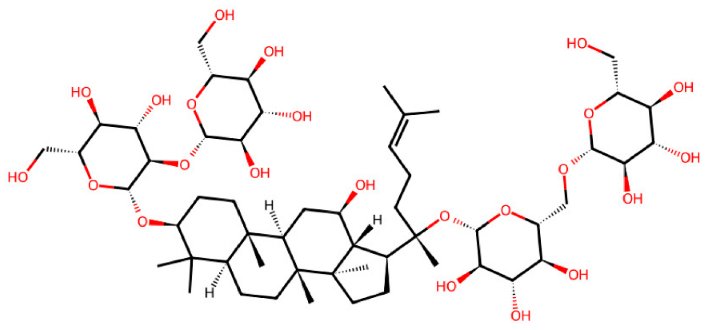	Panax ginseng	9898279
Tanshinone IIA	Diterpenoid (abietane)	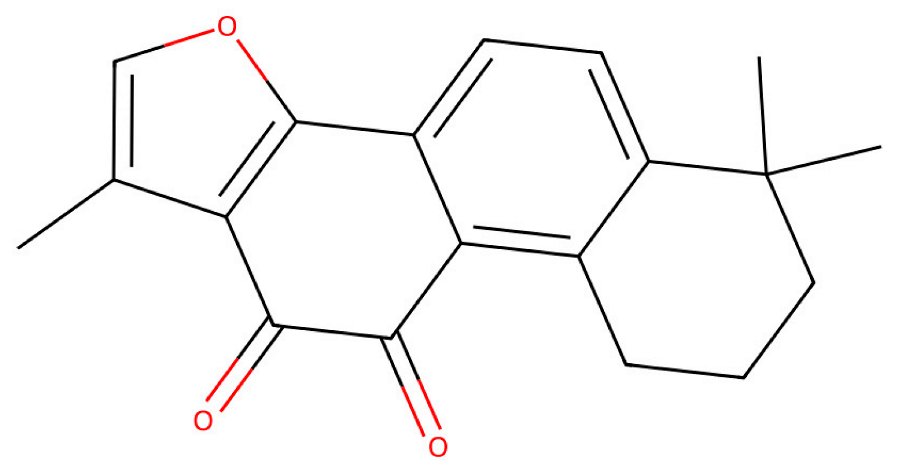	Salvia miltiorrhiza	164676
Curcumenol	Sesquiterpenoid	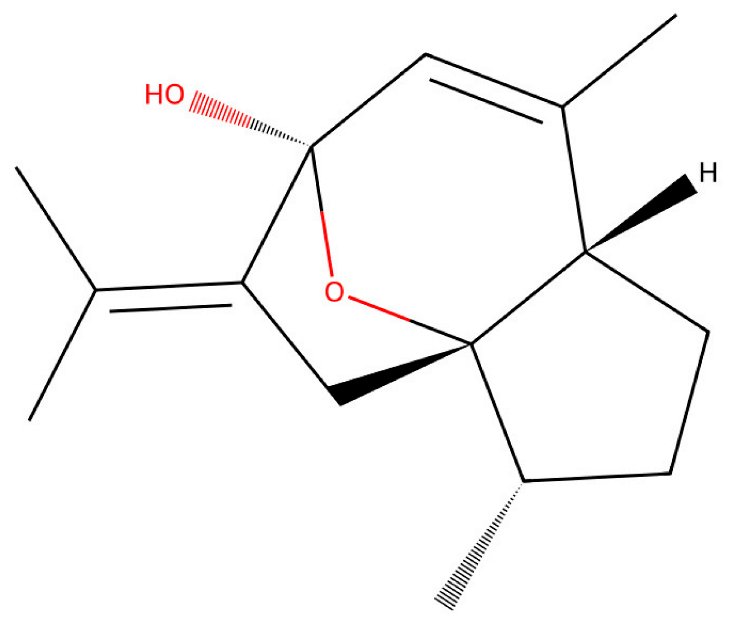	Curcuma species	167812
Elemene (β-elemene)	Sesquiterpenoid	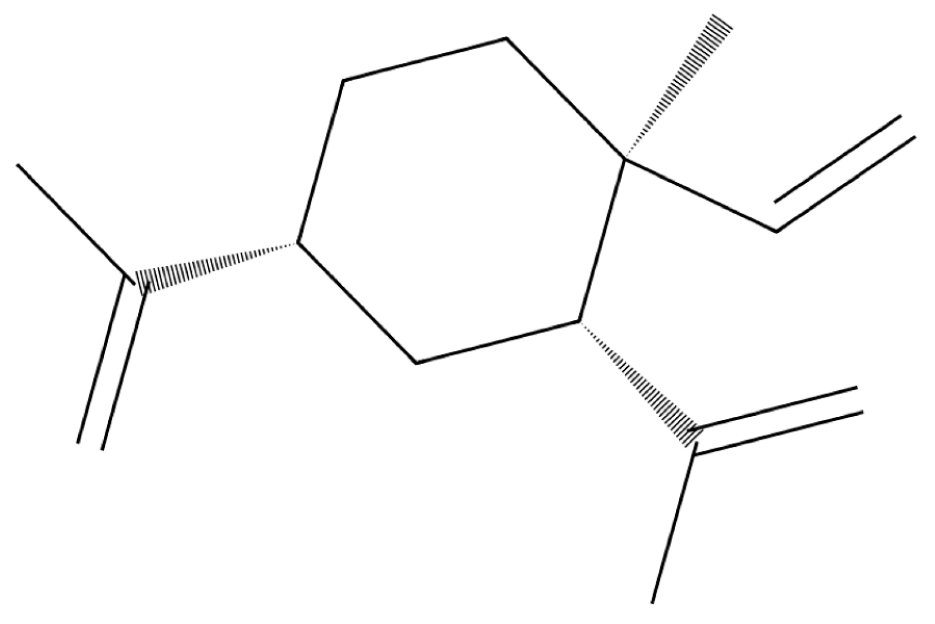	Curcuma species	6918391
Dihydroartemisinin (DHA)	Sesquiterpene lactone (endoperoxide)	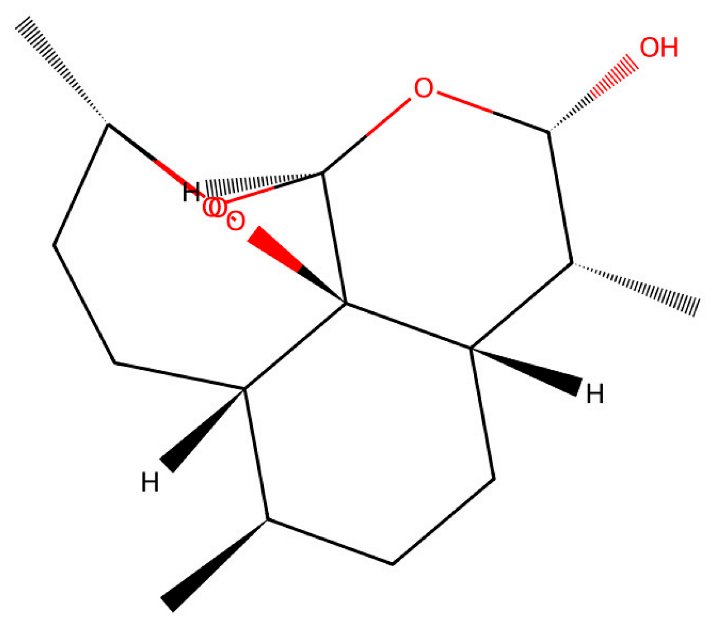	Artemisia annua	3000518
Gomisin M1	Lignan (dibenzocyclooctadiene)	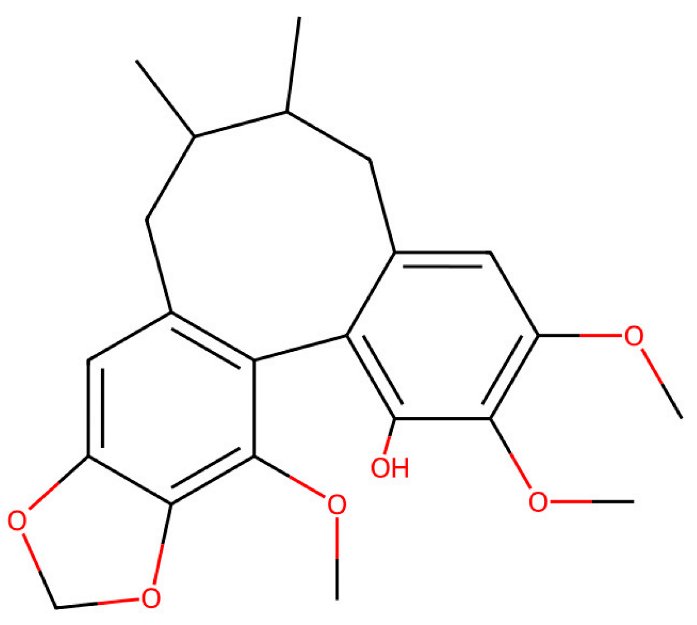	Schisandra chinensis	5321170
Berberine	Alkaloid (isoquinoline)	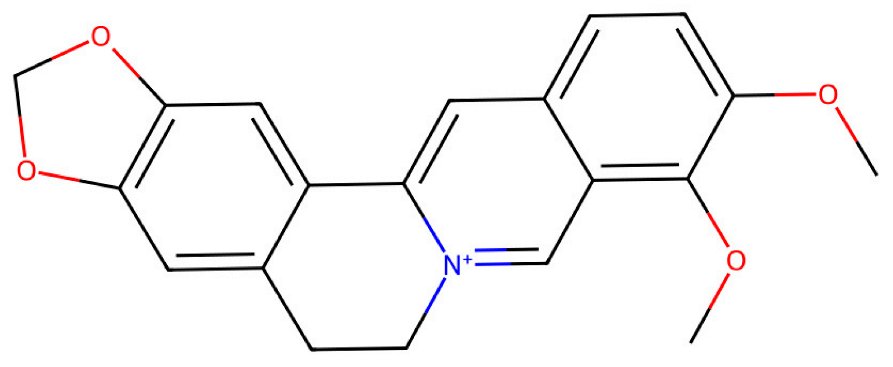	Coptis chinensis; Berberis	2353
Chelerythrine	Alkaloid (benzophenanthridine)	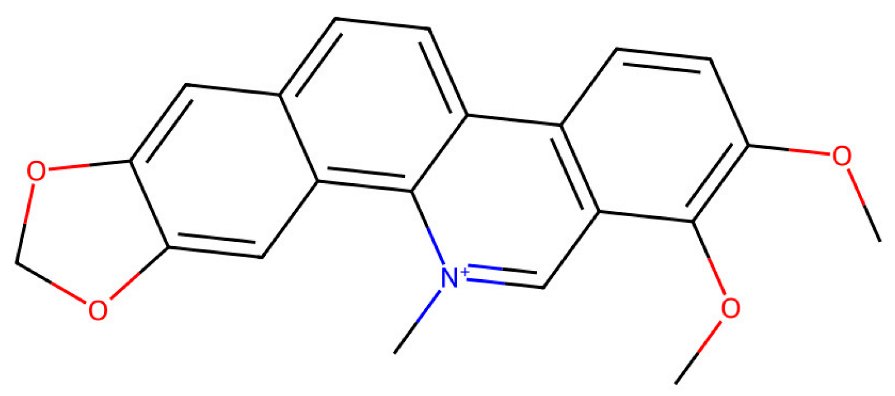	Chelidonium majus; Zanthoxylum	2703

## Data Availability

No new data were created or analyzed in this study. Data sharing is not applicable to this article.
